# Metacognitive team-based learning for creative problem-solving in Indian medical undergraduates: a single-arm pre-post quasi-experimental mixed-methods study

**DOI:** 10.1186/s12909-026-10157-2

**Published:** 2026-08-15

**Authors:** Sundip Hemant Charmode, Ciraj Ali Mohammed, Abdul Ajees Abdul Salam, Simmi Mehra, Sanjay Gupta

**Affiliations:** 1 Department of Anatomy, All India Institute of Medical Sciences (AIIMS) Rajkot, Para Pipaliya, Khanderi, Gujarat 360110 India; 2 College of Medicine and Health Sciences, National University of Science and Technology, Sohar, North Batinah 321 Oman; 3 Manipal Institute of Applied Physics, Manipal Academy of Higher Education, Manipal, Karnataka 576 104 India; 4 Department of Forensic Medicine and Toxicology, All India Institute of Medical Sciences (AIIMS) Rajkot, Para Pipaliya, Khanderi, Gujarat 360110 India

**Keywords:** Metacognition, Creative problem-solving, Medical education, Team-based learning, Undergraduate medical students, Mixed-methods, Anatomy education

## Abstract

**Background:**

Metacognitive and team-based strategies can strengthen problem-solving; however, many Indian medical undergraduates remain reliant on rote memorisation with limited exposure to structured, creativity-focused pedagogy. Structured interventions combining metacognitive training with collaborative, multidisciplinary teaching remain scarce in Indian undergraduate medical education. This study aimed to design, implement, and evaluate the Creative Problem-Solving Skills through Metacognition (CPSSM) module, a novel three-phase intervention for first-year MBBS students.

**Methods:**

A single-arm, pre-post, quasi-experimental, mixed-methods study was conducted at AIIMS Rajkot, India, during the 2024–2025 academic year. After obtaining ethical clearance and prospective trial registration, all 50 first-year MBBS students were recruited by universal sampling and 49 completed the intervention. Participants underwent three sequential phases: integrated teaching, case-based discussion, and collaborative model-making, delivered by a multidisciplinary faculty team. They were allocated to ten heterogeneous groups by stratified, non-random allocation based on baseline Metacognitive Awareness Inventory cut-off bands. The pre-specified primary outcome was post-intervention problem-solving accuracy, and the secondary outcomes included self-perceived creativity, group metacognition, and domain knowledge. Quantitative data were analysed using t-tests and ANOVA, with effect sizes and 95% confidence intervals. Qualitative feedback was analysed using descriptive content analysis informed by the Braun and Clarke thematic framework. No comparison group was included; therefore, the findings are reported as associations rather than causal effects.

**Results:**

Students with high baseline metacognitive awareness showed a significant within-group gain in problem-solving accuracy (28.59 to 30.87; mean difference = 2.28; *p* < 0.001; Cohen’s d = 1.53); the moderate-awareness group’s gain was smaller and non-significant. Female students outperformed males in problem-solving accuracy at both time points (pre-test: *p* < 0.01, d = 0.90). Self-perceived creativity was higher in the high metacognition group than in the moderate-metacognition group (59.09 vs. 52.72; *p* = 0.01). Thematic analysis identified teamwork, motivation, and growing confidence as the principal perceived gains, alongside coordination and resource-related challenges. Creativity was rarely cited spontaneously, despite being a primary intended outcome.

**Conclusions:**

Participation in the CPSSM module was associated with modest gains in problem-solving accuracy, which was most evident among students with stronger baseline metacognitive awareness. These findings support the feasibility of structured, metacognition-driven, team-based learning in early medical education; however, controlled, multi-site studies are needed before broader effectiveness can be claimed.

**Trial registration:**

Clinical Trials Registry–India, CTRI/2025/02/080559. This study was registered prospectively on February 14, 2025.

**Supplementary Information:**

The online version contains supplementary material available at https://doi.org/10.1186/s12909-026-10157-2.

## Introduction

India’s medical education system has undergone several commendable reforms in recent years, including the introduction of Competency-Based Medical Education (CBME), greater emphasis on applied learning, and early clinical exposure. These initiatives reflect a growing recognition of the need to align undergraduate training with modern healthcare demands. Globally, creative problem-solving and metacognitive skills are acknowledged as essential for cultivating adaptive, reflective, and competent health professionals [1–3]. However, national data reveal that significant challenges persist in fully integrating these skills into the Indian medical curriculum. National reports, including the National Institutional Ranking Framework (NIRF), together with published literature, indicate considerable variation in the implementation of undergraduate medical education across Indian institutions [1–5]. Although recent reforms, including CBME and early clinical exposure, represent important progress, many programmes continue to face challenges in embedding structured problem-solving, creative thinking, and active learning within routine teaching. Consequently, there is growing recognition of the need to strengthen learner-centred, problem-based, and metacognitive educational approaches to better prepare future medical graduates for the complexities of clinical practice [1–5]. These educational challenges extend beyond the classroom, as limitations in problem-solving and clinical reasoning may influence diagnostic accuracy and clinical decision-making [6]. Conversely, strong problem-solving skills have been associated with improved healthcare delivery, more effective resource utilisation, and enhanced patient safety [7].

Extensive global research demonstrates that students’ metacognitive awareness and regulation of their own thinking play a central role in creative problem-solving [8–10]. Students with strong metacognitive awareness are more adept at interpreting problems, selecting strategies, and monitoring their progress toward finding solutions. These skills are especially critical in healthcare, where ambiguity, complexity, and ethical dilemmas are routine occurrences. Studies from other disciplines, including arts, commerce, and STEM education, have highlighted that metacognitive abilities, combined with domain knowledge and motivation, are vital for solving open-ended and ill-structured problems [11, 12].

This study was guided by three a priori objectives: (i) to design and implement a structured metacognitive, multi-disciplinary, team-based module (Creative Problem-Solving Skills through Metacognition [CPSSM]) for first-year MBBS students; (ii) to evaluate pre–post changes in metacognitive awareness, problem-solving accuracy, self-perceived creativity, group metacognition, and domain knowledge; and (iii) to characterise learner-reported facilitators and barriers through qualitative feedback. The corresponding research question was: In a first-year MBBS cohort, is participation in the CPSSM module associated with measurable changes in these outcomes? The primary outcome was defined a priori as post-intervention problem-solving accuracy and all other comparisons were secondary or exploratory.

Although international interest in fostering creative problem-solving through metacognitive, team-based learning has increased, research in this area remains limited in Indian medical education, especially at the undergraduate level. To bridge this gap, the present study introduces the CPSSM module, a novel educational intervention initially implemented with first-year MBBS students, with faculty drawn from multiple disciplines (anatomy, clinical medicine, psychiatry, and allied health) involved in its design and delivery. The CPSSM module employs a quasi-experimental, mixed-methods design within a collaborative, team-based learning environment to evaluate how structured metacognitive strategies are associated with learners’ ability to address complex real-world problems creatively and critically. Because no parallel control or comparison group was included, pre-post changes are interpreted as associations rather than causal effects of the module, since improvements may also reflect testing effects, maturation, or familiarity with repeated assessments. While this study focuses on early stage learners, the module’s adaptable framework offers scalability across various phases of medical training, positioning it as a promising approach for cultivating metacognitive awareness and creative problem-solving throughout the continuum of medical education.

The module engages faculty from multiple disciplines (anatomy, surgery, medicine, orthopaedics, obstetrics and gynaecology, ophthalmology, ENT, and psychiatry) for two reasons. First, each case anchors an anatomical structure to a clinical pathology, which requires clinical specialty input at a curricular point when learners would otherwise encounter only basic science faculty. Second, metacognitive and group process elements require psychiatric and educational expertise that is unavailable within a single department. The intervention is therefore “multi-disciplinary faculty-led” rather than “interprofessional,” since learners are uni-professional (MBBS) while the teaching team is cross-disciplinary by design. The module is theoretically grounded in four complementary frameworks: (i) Flavell’s (1979) conceptualisation of metacognition as knowledge and regulation of one’s own cognitive processes [13]; (ii) Schraw and Dennison’s (1994) operationalisation of metacognitive awareness through knowledge-of-cognition and regulation-of-cognition components, which directly informed the assessment design [12]; (iii) constructivist, team-based learning theory, which positions structured group activity around authentic, ill-defined problems as a vehicle for higher-order cognitive development; and (iv) Braun and Clarke’s six-phase thematic analysis as the methodological frame for the qualitative strand.

## Methodology

### Study design and educational setting

This quasi-experimental, mixed-methods study was conducted during the 2024–2025 academic year in the Department of Anatomy at the All India Institute of Medical Sciences (AIIMS) Rajkot. AIIMS Rajkot is a tertiary-level medical institution recognised as an Institute of National Importance by the Government of India and offers undergraduate and postgraduate medical education. Although the primary implementation was led by the Department of Anatomy, faculty from clinical, psychiatric, and paramedical disciplines were actively involved in delivering the integrated teaching sessions. No parallel control or comparison group was included in this study. We used the SQUIRE-EDU, an extension of the SQUIRE 2.0 guidelines, to draft this manuscript, and the SQUIRE-EDU checklist when editing, included in Supplementary File S1 [14]. The study aimed to design, implement, and evaluate a multidisciplinary, faculty-led educational module focused on enhancing metacognitive awareness, collaborative problem-solving, creativity, and group metacognition among first-year MBBS students. As defined above, the module is multidisciplinary and faculty-led: the learners were drawn from a single profession (first-year MBBS students), while the faculty who designed and delivered the module were multidisciplinary. No parallel control or comparison group was included; pre-post changes were therefore reported as associations rather than as causal effects of the module, since improvements may also reflect testing effects of the module, maturation, or familiarity with repeated assessments.

### Ethical approval and trial registration

Ethical clearance was obtained from the Institutional Ethics Committee (IEC) of AIIMS Rajkot (Protocol ID: D/NF/74/2024; O.W. No.: AIIMS Rajkot/6th IEC/47/2025, dated 4th February 2025) [Supplementary File S2]. The study was prospectively registered on February 14, 2025, with the Clinical Trials Registry - India (CTRI) under the registration number CTRI/2025/02/080559 [Supplementary File S3].

### Participants and sampling

Universal sampling was employed. All 50 first-year MBBS students enrolled in the 2024–2025 batch were invited to participate in the study. Written informed consent was obtained from each student [Supplementary File S4]. One student was absent throughout the intervention, resulting in a final sample size of 49. All the questionnaires (MAI, SSCS, MCPS, GMS), along with the Model Grading Scale and the Module Feedback Questionnaire, were used to collect demographic and baseline data in addition to pre- and post-intervention data and are attached as Supplementary File S5.

#### Inclusion criteria

First-year MBBS students who provided informed consent.

#### Exclusion criteria

Students who declined consent or were absent for the entire intervention.

### Intervention: CPSSM module structure

The CPSSM module was developed by a multidisciplinary faculty team comprising anatomists, clinicians, psychiatrists, and allied health educators. The module consists of three sequential phases:

- *Phase 1 - Integrated Teaching Sessions:* Conducted in a lecture theatre, these sessions provided foundational knowledge for ten selected clinical case scenarios aligned with the anatomy curriculum.
- *Phase 2 - Case-Based Discussions:* Facilitated interdisciplinary discussions encouraged clinical reasoning, problem-solving, and contextual understanding.
- *Phase 3 - Collaborative Model-Making and Problem-Solving Task:* Conducted in the dissection hall, this phase involved hands-on group tasks in which students designed functional anatomical models representing their assigned clinical cases. Students were intentionally grouped into heterogeneous teams based on their baseline metacognitive scores, classified as low, moderate, or high, to promote balanced peer interaction and mutual scaffolding.

Each team was presented with an ill-defined real-world healthcare scenario that required critical thinking, stakeholder analysis, ethical reasoning, and interdisciplinary communication. The teams used guided templates to document their problem-solving approach, incorporating goal setting, strategic planning, and reflective checkpoints. The faculty emphasized creativity, anatomical accuracy, and collaboration, fostering deeper engagement with the learning material. The intervention was implemented over approximately four months (1st March – 30th June 2025). Students were divided into ten heterogeneous groups (nine groups of five and one group of four students) using stratified non-random allocation based on baseline MAI tertile scores (low < 25; moderate 26-39; high > 40). One to two students from each metacognitive level were assigned to each group to promote mutual scaffolding. These cut-off points were fixed thresholds adapted from prior MAI literature rather than tertiles derived from this sample’s own score distribution; applied to the present cohort, they yielded markedly uneven group sizes (low *n* = 1, moderate *n* = 25, high *n* = 23), as reported in Tables 3 and 4. The Principal Investigator (a faculty anatomist) led the Phase 1 and Phase 3 sessions, while clinician faculty and a psychiatrist facilitated the Phase 2 case-based discussions. The full multidisciplinary faculty team (anatomy, general surgery, ophthalmology, general medicine, psychiatry, orthopaedics, and allied health) contributed to module design and delivery. Each of the ten case-based modules followed an identical time structure: Phase 1 (Integrated Teaching Session) lasted 45 min, comprising gross anatomy teaching (20 min), applied/clinical-correlation teaching delivered by faculty from the relevant clinical department (15 min), and application of metacognitive skills and strategies (10 min); Phase 2 (Case-Based Discussion) lasted 10 min, built around a single clinical vignette per topic. Phases 1 and 2 together therefore totalled 55 min per case topic (approximately 9 h and 10 min cumulatively across all ten topics). Phase 3 (the model-making task) was conducted in a separate session in the dissection hall. Ten clinical cases were selected to span major organ systems and regions of the first-year MBBS anatomy curriculum (hepatobiliary, inguinal/abdominal wall, pelvic floor, lower limb vascular/joint anatomy, foot biomechanics, cranial nerve, and neuroanatomical/ENT topics), with each case built around a common, clinically recognisable pathology linked to a core anatomical structure (e.g., portal hypertension for liver anatomy, inguinal and femoral hernia for the inguinal canal and femoral triangle, pelvic organ prolapse for the pelvic diaphragm, flat foot for the arches of foot, anterior cruciate ligament injury for the knee joint, Bell’s palsy for the facial nerve, hydrocephalus for the ventricular system, vocal cord palsy for the larynx, and otitis media for the tympanic cavity). Each case was further selected for its suitability for physical/structural model representation and for requiring input from a correlating clinical department, ensuring the integration of anatomical, clinical, and problem-solving learning objectives within a single case. A representative Phase 2 vignette (Module 1, Liver/Portal Hypertension) is as follows: “A 52-year-old male with chronic alcohol use presents with abdominal swelling, vomiting blood, and fatigue. Examination revealed ascites, splenomegaly, and oesophageal varices. Investigations confirmed portal vein dilation. What is the likely diagnosis and differential diagnosis? Mention appropriate investigations and likely complications if untreated.” The complete set of ten cases, including module aims, framework, session plans, and rubrics, is provided in Supplementary File S6.

**Fig. 1:**
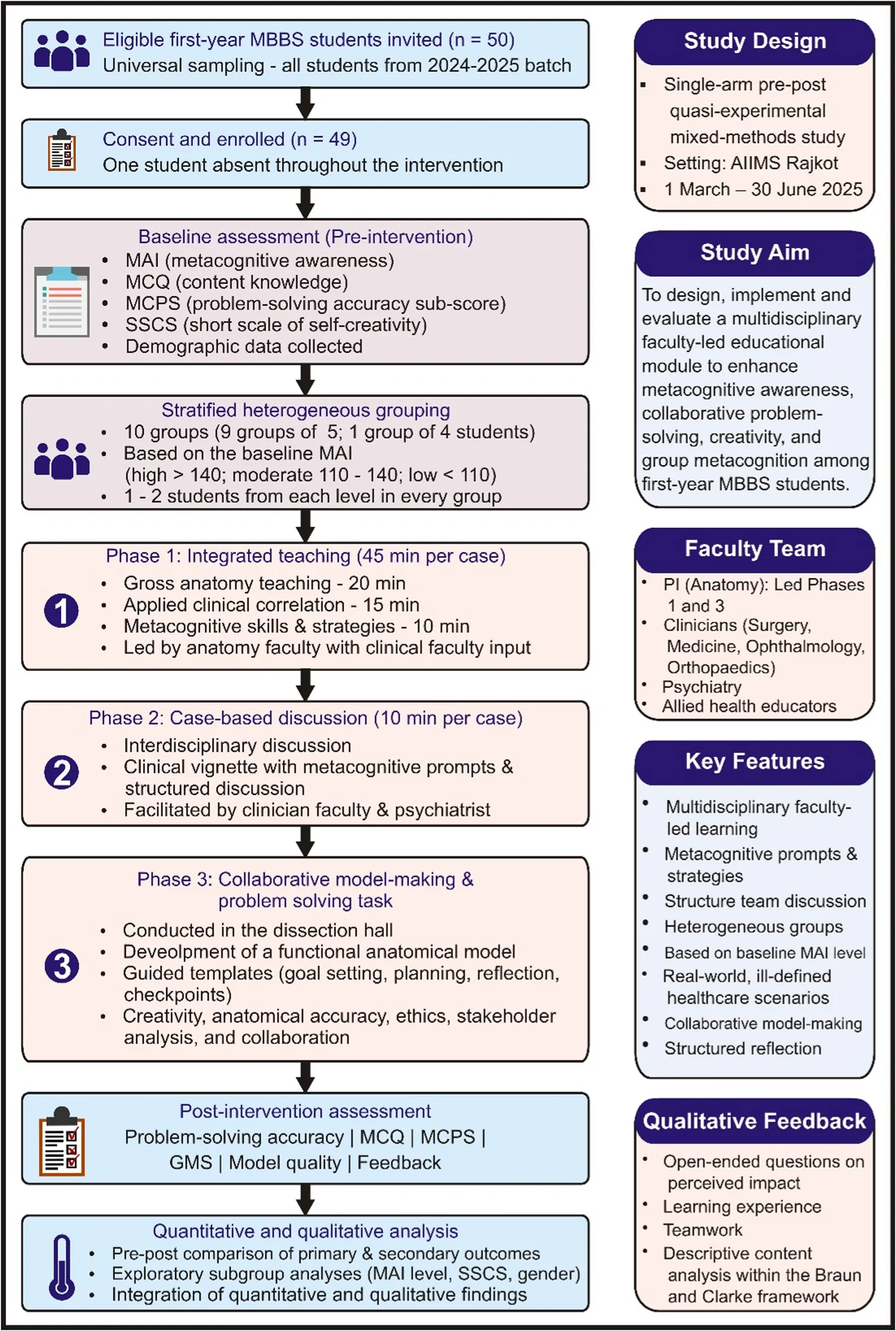
Study design and workflow of the CPSSM module. Eligible first-year MBBS students were recruited by universal sampling and completed baseline assessments before being allocated to heterogeneous groups based on baseline metacognitive awareness. The intervention comprised three sequential phases: integrated teaching, case-based discussion, and collaborative model-making. Post-intervention assessments included problem-solving accuracy, knowledge, metacognition, group metacognition, model performance, and learner feedback. Quantitative and qualitative data were analysed independently and integrated during interpretation using a convergent parallel mixed-methods approach

Four principles guided module design: (i) alignment with the first-year MBBS anatomy syllabus, so participation did not compete with curricular load; (ii) authentic, ill-defined clinical problems requiring integration rather than recall; (iii) a three-phase progression (integrated teaching → case discussion → collaborative model-making) of escalating cognitive load; and (iv) stratified heterogeneous grouping by baseline MAI tertile, treating metacognitive variation as a designed scaffold rather than a confounder. Model-making rubric scores were assigned through blinded scoring by an independent faculty rater, separate from the qualitative coding team, yielding an inter-rater intraclass correlation coefficient of 0.88 against a second scorer. A schematic flow diagram of the study design (baseline assessment → Phase 1 → Phase 2 → Phase 3 → post-intervention assessment) is provided in Fig. 1 to visually anchor the design.

### Assessment tools and data collection methods

#### Pre-intervention assessments

Before implementing the intervention, baseline assessments were conducted to evaluate metacognitive awareness, domain knowledge, and problem-solving accuracy. Metacognitive awareness was assessed using a 52-item adapted version of the Metacognitive Awareness Inventory (MAI), originally developed by Schraw and Dennison (1994), with appropriate copyright permissions obtained for adaptation and reuse [12]. Domain knowledge was measured using a 20-item multiple-choice questionnaire (MCQ) administered via Google Forms, focusing on anatomy-related clinical concepts. Problem-solving accuracy was operationalised as a 10-item sub-score (items 1–10) of the Metacognition in Creative Problem-Solving Scale (MCPS), scored out of 50 [6, 14, 15]. Participants’ self-perceived creativity was assessed using a modified 11-item version of the Short Scale of Self-Creativity (SSCS), yielding a cumulative score with a maximum attainable value of 77 [17]. For clarity, two related but distinct constructs are referred to in this manuscript. “Problem-solving accuracy” refers to the MCPS items 1–10 sub-score (scored out of 50), assessed at both pre-test and post-test; it is treated as the primary outcome of this study and is distinct from the separate model-making grading rubric (11 criteria, 115 marks; Supplementary File S6) used to evaluate the physical models themselves. “Creative problem-solving” refers to the broader cognitive process of generating novel, contextually appropriate solutions to ill-defined problems, which is the construct that the MCPS scale is designed to capture and which the module aims to develop. The two constructs are related (creative problem-solving is one route to accurate solutions in ill-defined cases) but are measured by different instruments and reported separately in the Results section.

#### Post-intervention assessments

Following the intervention, outcome assessments were conducted to measure the impact of the educational module. Model performance was evaluated using a structured, study-specific grading rubric covering 11 criteria: anatomical accuracy, functionality/mechanics, creativity and innovation, clinical relevance, teamwork and collaboration, problem-solving application, metacognitive reflection, presentation clarity, model durability, use of materials, and overall learning impact, scored out of 115 (full rubric in Supplementary File S6) [6, 7]. Knowledge reassessment was conducted using a second 20-item MCQ consisting of new, conceptually equivalent questions to the pre-test, which was also delivered via Google Forms. Problem-solving accuracy was reassessed post-intervention using the same 10-item MCPS sub-score (items 1–10), scored out of 50 points [6, 14, 16]. To measure students’ strategic thinking, the application of metacognitive strategies was assessed using an 11-item MCPS adapted from the original MAI framework [6, 15]. Group metacognition was evaluated using a 27-item Group Metacognition Scale (GMS) specifically developed for collaborative learning contexts and derived from the MAI [18]. Finally, student feedback was collected using a module feedback questionnaire comprising Likert-scale items (rated 1–5) and open-ended questions designed to capture qualitative reflections on their learning experience and the perceived value of the intervention [19]. A separate, formally validated faculty observation checklist was not employed in this iteration of the module; faculty assessment of student performance during Phase 3 was instead captured through the structured model-making grading rubric described above, and this is noted as a direction for future refinement in the Limitations. Table 1 summarizes the standardized instruments used for pre- and post-intervention assessments.

**Table 1:** Summary of Standardized Instruments Used for Pre- and Post-Intervention Assessments

Instrument	Construct	Items	Score	Source	Evidence
MAI	Metacognitive awareness	52	0–52	Adapted [12]	Parent α = 0.93; in-sample α not computed
Knowledge MCQ (Pre)	Anatomy knowledge	20	0–20	Original	Content validated
Knowledge MCQ (Post)	Anatomy knowledge	20	0–20	Original	Parallel form
MCPS Problem-solving Accuracy	Problem-solving accuracy	10	10–50	Adapted [6]	Parent α = 0.87
SSCS	Self-perceived creativity	11	11–77	Adapted [17]	Parent α = 0.88
Model Rubric	Model performance	11	0–115	Original	ICC = 0.88
MCPS	Metacognitive strategies	11	11–55	Adapted [6]	Parent α = 0.87
GMS	Group metacognition	27	27–135	Adapted [18]	Parent α = 0.91
Feedback Questionnaire	Learner perceptions	Likert + OE	1–5	Original	Faculty reviewed

*α* Cronbach’s alpha, *ICC* intraclass correlation coefficient, *MCQ* multiple-choice questionnaire. Published reliability coefficients of the parent instruments are reported because in-sample reliability was not re-computed for this cohort; this is acknowledged as a methodological limitation. The model-making grading rubric and module feedback questionnaire were study-specific instruments reviewed for content validity by the multidisciplinary faculty team. Full descriptions of the intervention materials, grading rubric, questionnaires, and assessment tools are provided in Supplementary File S6. Numbers in square brackets refer to the reference list

#### Data collection logistics

Data were collected using a combination of online and offline methods. Google Forms were used for all the MCQ-based assessments and feedback collection. Mobile phones facilitated online submissions, while offline activities, such as model construction and observations, were supported with printed templates and structured rubrics. Stationery and low-cost materials were provided during the model-making phase. All responses, including quantitative scores and qualitative reflections, were compiled into a master dataset in Excel for further analysis.

#### Data analysis

Quantitative data were analysed using IBM SPSS Statistics version 25.0 [20]. The primary outcome was post-intervention problem-solving accuracy. Descriptive (means and standard deviations) and inferential statistics (paired t-tests) were used to compare pre- and post-intervention scores. Pre–post comparisons of the primary outcome (problem-solving accuracy) and student-reported perceptions of learning constituted the principal analyses. Subgroup comparisons, including gender, baseline metacognitive level, and SSCS category, are exploratory and were not pre-specified as hypothesis-driven tests; they are reported descriptively to inform future adequately powered studies. Statistical significance was set at *p* < 0.05. Subgroup analyses explored the associations between baseline metacognitive levels, gender, and key outcome metrics. Qualitative data from open-ended feedback and reflection components were analysed using thematic analysis following Braun and Clarke’s six-phase approach. The content was categorized into themes such as metacognitive development, problem-solving, creativity, teamwork, and collaborative learning. To ensure coding reliability, independent coding was performed by multiple raters. The principal investigator and two external clinician educators independently coded a random 20% subset of responses, achieving an initial agreement of 82%. Discrepancies were resolved through discussion to reach a consensus, yielding a final Cohen’s kappa of 0.87. The internal consistency of the multi-item scales was not separately re-computed in the present sample, which is acknowledged as a methodological limitation. Previously reported reliabilities for the parent instruments are as follows: MAI Cronbach’s α = 0.93 [12]; SSCS α = 0.88 [17]; MCPS α = 0.87 [6]; GMS α = 0.91 [18]. Adaptations of the MAI, SSCS, problem-solving questionnaire, and MCPS were limited to wording changes contextualising items to anatomy-related clinical scenarios, with no items added or removed except for the modified 11-item SSCS; all rewording was reviewed by the multidisciplinary faculty team. The model-grading rubric and feedback questionnaire were study-specific and content-reviewed by faculty; formal construct validity was not separately re-examined and is acknowledged in the limitations. Given the multiple subgroup comparisons (gender, baseline metacognitive level, SSCS category) and to address the risk of Type I error, post-intervention problem-solving accuracy was designated a priori as the sole primary outcome; all other comparisons were reported as secondary or exploratory and were not formally adjusted for multiple testing, with this exploratory framing made explicit in the Results and Discussion sections. Effect sizes (Cohen’s d for two-group comparisons; eta-squared for ANOVA) and 95% confidence intervals were reported alongside p-values. Assumptions for parametric tests (normality via Shapiro–Wilk; homogeneity of variance via Levene’s test) were checked prior to t-tests and ANOVA; non-parametric alternatives (Mann–Whitney U, Wilcoxon signed-rank) were used where assumptions were violated and are flagged in the Results. The mixed-methods design is best described as a convergent parallel design: quantitative and qualitative data were collected during the same intervention window, analysed independently, and integrated at the interpretation stage by examining whether learner-reported facilitators and barriers converged with, complemented, or diverged from the pre–post quantitative findings.

## Results

To evaluate the outcomes associated with the CPSSM educational intervention, quantitative and qualitative data were collected from 49 first-year medical students. The quantitative analysis focused on measuring changes in metacognitive awareness, collaborative learning, and problem-solving accuracy before and after the intervention, while also examining subgroup differences by gender, baseline metacognitive awareness, and collaborative skills. In parallel, qualitative reflections were gathered through open-ended feedback to capture students’ experiences, perceived benefits, and challenges associated with the collaborative model-making task. This mixed-methods approach allowed for a comprehensive understanding of the cognitive and experiential impact of the intervention.

### Quantitative data

**Fig. 2:**
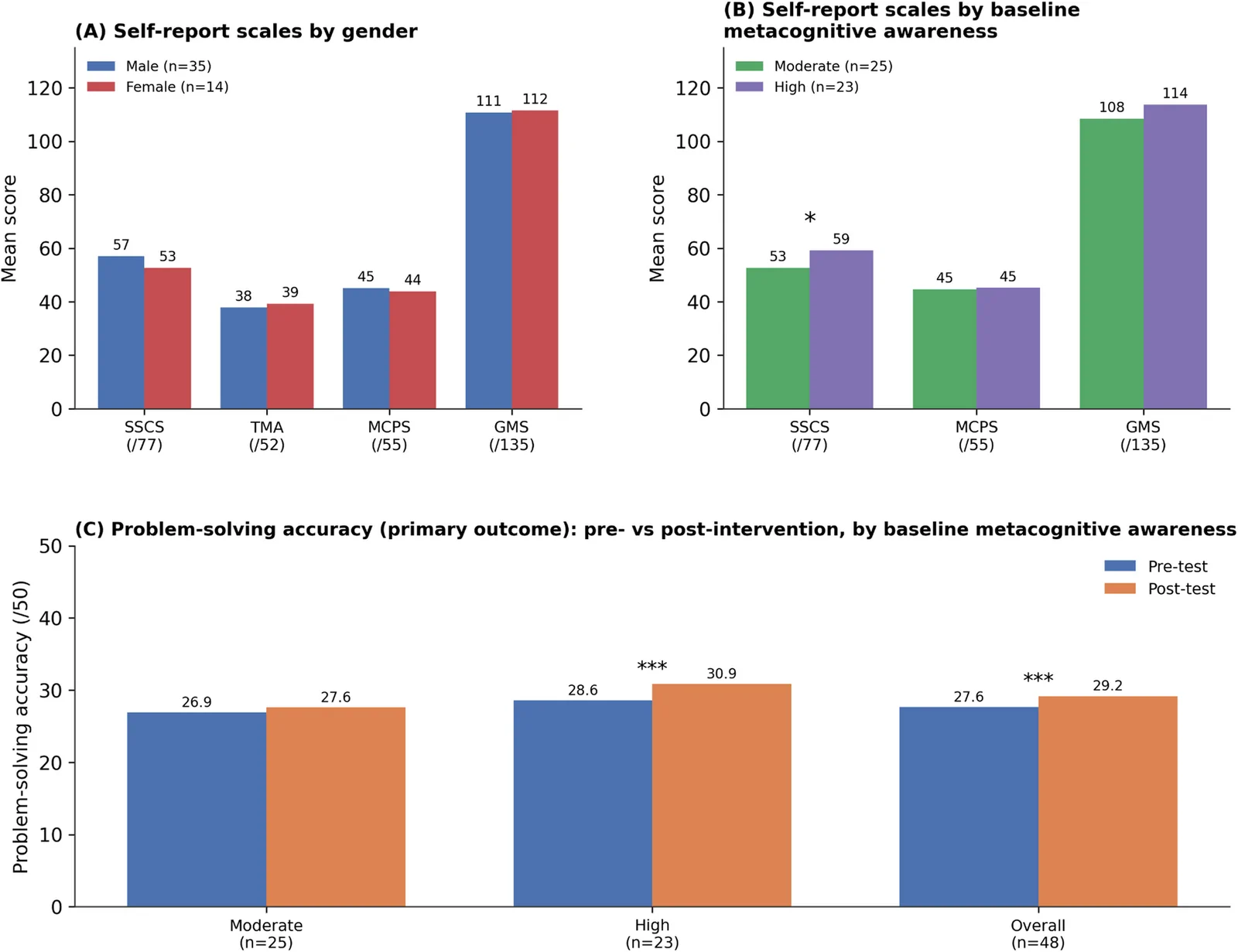
Quantitative comparison of self-report scales and primary outcomes. (**A**) Mean self-report scale scores by gender (Male, *n* = 35; Female, *n* = 14). (**B**) Mean self-report scale scores by baseline metacognitive awareness level (Moderate, *n* = 25; High, *n* = 23). (**C**) Problem-solving accuracy (primary outcome) before and after the intervention, by baseline metacognitive awareness level and overall. Each scale is plotted against its own maximum (shown in parentheses on the x-axis labels); the scales are not directly comparable to one another in absolute terms. *Note.* Comparisons in (**A**) and (**B**) by independent-samples t-test; within-group pre–post change in (**C**) by paired-samples t-test. * *p* < 0.05; *** *p* < 0.001. The *N* = 48 analyses in (**B**) and (**C)** exclude the single participant in the low metacognitive awareness category (*n* = 1), who could not form a comparison group. Objective knowledge scores are not shown here as they were not a self-reported creativity/metacognition outcome of this analysis

The quantitative findings were presented through a series of comparative analyses across gender, metacognitive awareness levels, and collaborative skill categories. These analyses examined both pre- and post-intervention performance and changes in key outcome variables (Tables 2, 3 and 4 and Fig. 2).

**Table 2:** Gender-wise Comparison of Metacognitive and Problem-Solving Metrics (*N* = 49)

Variable	Max Score	Gender	*N*	Mean (SD)	t-value	*p*-value
SSCS Score	77	Male	35	56.97 (9.35)	1.48	0.14
		Female	14	52.71 (8.20)		
Knowledge about Cognition	17	Male	35	12.37 (2.40)	0.21	0.83
		Female	14	12.21 (2.19)		
Regulation of Cognition	35	Male	35	25.43 (4.83)	1.04	0.30
		Female	14	27.00 (4.57)		
Total Metacognitive Awareness	52	Male	35	37.80 (5.88)	0.77	0.44
		Female	14	39.21 (5.56)		
Problem-Solving Accuracy (Pre-test)	50	Male	35	25.21 (10.28)	**2.84**	**< 0.01**
		Female	14	33.75 (7.05)		
Problem-Solving Accuracy (Post-test)	50	Male	35	27.00 (10.88)	**2.36**	**0.02**
		Female	14	34.46 (7.15)		
Group Metacognition Score	135	Male	35	110.77 (11.21)	0.22	0.82
		Female	14	111.57 (11.06)		
MCPS Score	55	Male	35	45.11 (3.78)	1.06	0.29
		Female	14	43.93 (2.76)		

Comparisons using independent-samples t-test. Bolded p-values indicate statistical significance (*p* < 0.05). *SSCS* Short Scale of Creativity-Self, *MCPS* Metacognition in Creative Problem-Solving Scale, *SD* standard deviation

**Table 3:** Comparison of Creativity, Problem-Solving Accuracy, Metacognition, and Objective Knowledge Pre- and Post-Test Scores between Participants with Moderate and High Levels of Baseline Metacognitive Awareness (*N* = 48)

Variable	Max Score	Metacognition Level	*N*	Mean (SD)	t-value	*p*-value
SSCS Score	77	Moderate	25	52.72 (10.55)	**2.51**	**0.01**
		High	23	59.09 (6.30)		
Problem-Solving Accuracy (Pre-test)	50	Moderate	25	26.90 (11.49)	0.56	0.57
		High	23	28.59 (8.95)		
Problem-Solving Accuracy (Post-test)	50	Moderate	25	27.60 (11.67)	1.07	0.28
		High	23	30.87 (9.16)		
MCPS Score	55	Moderate	25	44.64 (3.72)	0.52	0.59
		High	23	45.17 (3.23)		
Group Metacognition Score	135	Moderate	25	108.48 (11.70)	1.65	0.10
		High	23	113.74 (10.11)		
Objective Knowledge (Pre-test)	20	Moderate	25	13.96 (1.88)	0.52	0.60
		High	23	14.22 (1.48)		
Objective Knowledge (Post-test)	20	Moderate	25	13.24 (2.82)	1.22	0.22
		High	23	14.09 (1.81)		

Comparisons using independent-samples t-test. Bolded p-values indicate statistical significance (*p* < 0.05). The baseline metacognitive awareness tertiles were yielded low (*n* = 1), moderate (*n* = 25), and high (*n* = 23). The single participant in the low category was excluded from this between-category comparison because a one-person group cannot be statistically compared, resulting in *N* = 48

*SSCS* Short Scale of Self-Creativity, *MCPS* Metacognition in Creative Problem-Solving Scale, *SD* standard deviation

**Table 4:** Problem-Solving Accuracy by SSCS Categories and Metacognitive Awareness Level

SSCS categories (post-test only)						
Group	N	Pre-test Mean (SD)	Post-test Mean (SD)	Mean Difference (MD)	Test statistic	*p*-value
Low	5	–	28.00 (13.16)	–	F = 0.35	0.70
Moderate	38	–	28.75 (10.59)	–		
High	6	–	32.50 (8.06)	–		
Total	49	**–**	29.13 (10.45)	**–**		

*Metacognitive Awareness (paired pre–post within group)*						
Group	N	Pre-test Mean (SD)	Post-test Mean (SD)	Mean Difference (MD)	Test statistic	*p*-value
Moderate	25	26.90 (11.49)	27.60 (11.67)	0.70	t = 1.49	0.148
High	23	28.59 (8.95)	30.87 (9.16)	2.28	t = 7.34	< 0.001
Between-group (post-test only)	48	–	–	–	t = 1.07	0.28
Total	48	27.65 (10.17)	29.13 (10.45)	1.48	t = 4.93	< 0.001

The SSCS-category analysis (post-test only) included the full sample (*N* = 49) and was assessed using one-way ANOVA. The Metacognitive Awareness analysis reflects *N* = 48 because the single participant in the low metacognitive awareness category (*n* = 1) was excluded from the between-category comparison, as a one-person group cannot be statistically compared; within-group pre–post change was assessed by paired-samples t-test, and the between-group post-test difference by independent-samples t-test. Bolded values indicate statistical significance (*p* < 0.05). Dashes (–) indicate data that are not applicable or not collected for that cell. *MD* mean difference, *SD* standard deviation. The *N* = 48 and *N* = 49 post-test totals coincide because the excluded low-MAI participant’s score equals the overall mean

#### Gender-wise comparison of cognitive and problem-solving metrics

Table 2 presents a comparison of various cognitive and problem-solving metrics between male and female participants using independent sample t-tests. Statistically significant gender differences were observed in problem-solving accuracy scores both before and after the intervention. In the pre-test, female students (M = 33.75, SD = 7.05) scored significantly higher than male students (M = 25.21, SD = 10.28), t(47) = 2.84, *p* < 0.01 (exact two-tailed *p* ≈ 0.0066), Cohen’s d = 0.90, 95% CI [2.49, 14.59] for the mean difference. Similarly, in the post-test, female students maintained their performance advantage (M = 34.46, SD = 7.15 vs. M = 27.00, SD = 10.88 for males), t(47) = 2.36, *p* = 0.02 (Cohen’s d = 0.75, 95% CI [1.10, 13.82] for mean difference). These findings suggest that, although the intervention benefited both groups, female students consistently demonstrated stronger individual problem-solving accuracy. In contrast, there were no statistically significant sex differences in the SSCS score, knowledge and regulation of cognition, total metacognitive awareness, group metacognition, or MCPS scores (*p* > 0.05 for all). These results indicate that both genders were comparably equipped in terms of their metacognitive and collaborative capacities.

#### Differences based on baseline metacognitive awareness

Table 3 compares collaborative and cognitive metrics between students with moderate and high baseline metacognitive awareness. A statistically significant difference was observed in SSCS scores, where students in the high metacognitive group scored higher (M = 59.09, SD = 6.30) than those in the moderate group (M = 52.72, SD = 10.55; t(46) = 2.51, *p* = 0.01). This suggests that students with stronger metacognitive abilities are more likely to perceive themselves as effective. No significant differences were found between the two groups in terms of problem-solving accuracy (pre- or post-test), MCPS scores, group metacognition, or objective knowledge assessments (*p* > 0.05).

#### Problem-solving accuracy by SSCS and metacognitive category

Table 4 examines post-test problem-solving accuracy across SSCS and metacognitive awareness categories. Table 4 shows that no significant differences existed in post-test scores across SSCS levels (low, moderate, high), F(2, 46) = 0.35, *p* = 0.70. Although high-SSCS students had slightly better mean scores (M = 32.50), this was not statistically significant. Table 4 compares the post-test scores between the moderate and high metacognitive awareness groups. Students with high awareness performed slightly better (M = 30.87 vs. 27.60), but this difference was not significant, t(46) = 1.07, *p* = 0.28.

#### Pre–post improvements in problem-solving accuracy

Table 4 presents pre- and post-test comparisons by metacognitive group. Students in the high-awareness group showed a statistically significant improvement (MD = 2.28, *p* < 0.001), whereas the moderate group’s gain was smaller and not significant (MD = 0.70, *p* = 0.148). Combined, all students showed a meaningful increase in scores (MD = 1.48, *p* < 0.001), suggesting an association between participation in the intervention and improved problem-solving accuracy, particularly for students with higher baseline metacognitive awareness. Figure 2 presents a bar chart displaying the comparative analysis of self-creativity, metacognitive awareness, and problem-solving accuracy among, the study participants.

#### Qualitative data

Table 5 summarizes students’ open-ended responses regarding the factors that facilitated or hindered their model-making experience. The most frequently cited facilitator was teamwork (63.27%), followed by motivation (18.37%), knowledge (12.24%), and teamwork combined with creativity (6.12%). These responses highlight the importance of collaborative engagement and group dynamics. The reported challenges included lack of coordination (20.41%), lack of resources, limited model-building judgment, and gaps in structural knowledge (each 18.37%). Other concerns included a lack of simulation knowledge (16.33%), time constraints (6.12%), and limited creativity (2.04%). These qualitative findings underscore the benefits and practical challenges of implementing team-based active learning in foundational medical science courses.

**Table 5:** Students’ Reported Facilitators and Challenges in Completing the Model-Making Task (*N* = 49)

Sl. No.	Facilitators	Frequency	(%)	Sl. No.	Challenges	Frequency	(%)
1	Teamwork	31	63.27	1	Lack of Coordination Between Members	10	20.41
2	Motivation	9	18.37	2	Lack of Resources	9	18.37
3	Knowledge	6	12.24	3	Lack of Judgement About Model-Making	9	18.37
4	Teamwork and Creativity	3	6.12	4	Lack of Knowledge of Structure	9	18.37
				5	Lack of Knowledge About Simulation	8	16.33
				6	Time Constraints	3	6.12
				7	Lack of Creativity	1	2.04

Responses were short-answer codes from an open-ended feedback questionnaire; the percentages reflect the proportion of the 49 respondents citing each coded category. Facilitators were reported across four categories and challenges across seven; blank facilitator cells were structural and did not indicate missing data

**Fig. 3:**
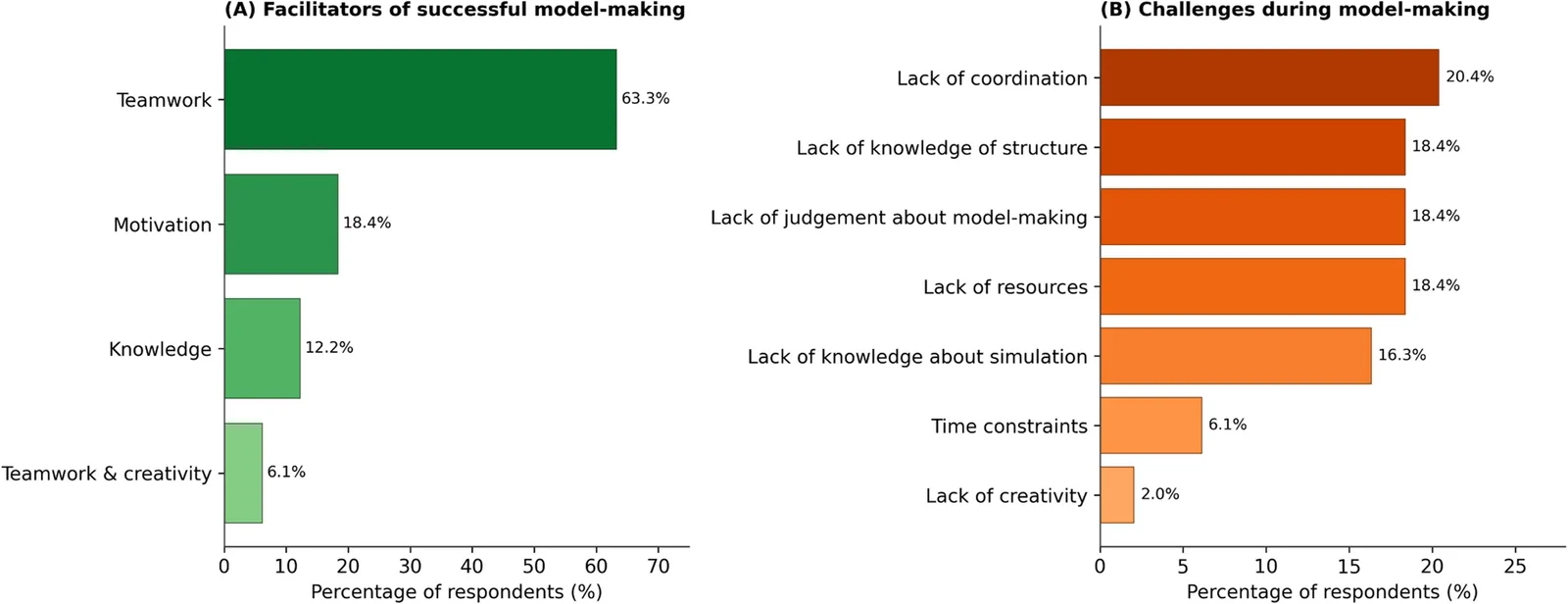
Descriptive content analysis of student reflections on the model-making task (*N* = 49). (**A**) Factors that students reported as facilitating successful completion of the activity. (**B**) Challenges reported by the students reported during the activity. The bars show the percentage of respondents citing each coded category. *Note.* Responses were short-answer codes from an open-ended feedback questionnaire; a single response could map to one dominant category, and percentages are of the 49 respondents. Facilitators comprised four coded categories and challenges seven

Table 5 presents a descriptive frequency/content analysis of the open-ended responses, distinct from the full Braun and Clarke thematic analysis described in the Data Analysis section. Representative participant comments illustrating the dominant facilitator theme included statements emphasizing peer support and shared problem-solving during the model-making task. Because the open-ended items were captured as short-answer responses rather than the extended reflections, participant comments most often took the form of single dominant terms. The most frequently used learner-supplied words for what helped them complete the task were “teamwork” (*n* = 31) and “motivation” (*n* = 9), with three students explicitly combining “teamwork and creativity”; for challenges, the most common terms were “coordination” (*n* = 10), “resources” (*n* = 9), and “knowledge of the structure of the model” (*n* = 9). When asked what they had learned from the module, the most common student-supplied responses were “teamwork and collaborative skills” (*n* = 20), “creativity skills” (*n* = 9), “confidence and presentation skills” (*n* = 9), and “problem-solving skills” (*n* = 8). One participant qualified their willingness to repeat the experience with the comment “yes, but with less frequency.” On a separate open-ended item asking about the module’s effect on routine academic workload, several students reflected on workload effects with comments such as “academics got affected” (*n* = 11) and “time constraints for model making” (*n* = 8). Read alongside the quantitative findings, these reflections are broadly consistent with the pattern of modest, metacognition-linked gains reported above: students most often credited collaborative processes (teamwork, motivation) rather than the creativity-focused activities for their learning, suggesting that the qualitative and quantitative strands converge on collaboration as a stronger driver of engagement than of measurable problem-solving gain, while diverging on the relatively limited spontaneous mention of creativity despite it being a primary intended outcome of the module. Figure 3 displays the facilitators and barriers perceived by students during the model-making activity.

### Summary of results

Quantitative analysis revealed that female students significantly outperformed male students in both pre- and post-test problem-solving assessments, while showing similar levels of metacognitive and collaborative skills. Students with high metacognitive awareness not only had better collaborative skills but also showed significantly greater gains in post-intervention problem-solving accuracy. However, no significant differences were observed in post-test scores based on SSCS levels or metacognitive categories. Qualitative feedback emphasized teamwork and motivation as key drivers of engagement, whereas coordination difficulties and knowledge/resource gaps were notable challenges. As this was a single-arm, pre–post design without a comparison group, these findings are associative: they suggest that the CPSSM intervention may be linked to gains in problem-solving accuracy, particularly for students with strong metacognitive foundations, but do not establish that the module caused these improvements.

## Discussion

This study explored the interrelationship between metacognitive awareness, collaborative engagement, and problem-solving accuracy among first-year medical students following a structured educational intervention. The findings presented in Table 2 reveal noteworthy gender-based patterns. Female students significantly outperformed their male counterparts in both pre- and post-test problem-solving assessments, suggesting a consistent cognitive advantage. These results are consistent with the existing literature, which attributes such trends to the more reflective and organized learning strategies often employed by female learners in academic settings (Zohar & Dori, 2003; Voyer & Voyer, 2014) [21, 22]. However, these differences were not mirrored in metacognitive awareness, MCPS, or collaborative skills, indicating that while the underlying cognitive processes were similar across genders, the application of those processes in performance contexts may vary. Notably, the absence of significant gender differences in the SSCS and MCPS suggests that both male and female learners perceived themselves to be comparably collaborative and reflective. The slight advantage in SSCS scores among males, though statistically non-significant, invites further inquiry into how gendered perceptions of collaboration manifest and influence team-based learning in the future.

Table 3 emphasized the influence of baseline metacognitive awareness on collaborative performance. Students with high metacognitive awareness exhibited significantly greater SSCS scores than their moderately aware peers, reinforcing the idea that metacognitively attuned learners are more strategic in group contexts. They likely engage more purposefully with task goals, seek feedback proactively, and regulate group processes more effectively behaviours that enhance collective performance (Schraw, 1998; Veenman et al., 2006) [23, 24]. However, this elevated collaborative competence did not translate into statistically significant differences in individual problem-solving or knowledge scores. This may reflect the need for sustained engagement with metacognitive strategies over time to produce measurable gains in individual cognitive domains or the possibility that short-term interventions, such as the CPSSM, primarily shape attitudes and behaviours rather than deeply embedded cognitive performance.

Interestingly, the data in Table 3 suggest that a higher perceived collaborative ability (SSCS) alone does not lead to significantly better individual problem-solving outcomes. Although students in the high SSCS group performed slightly better in the post-test assessments, the differences were not statistically significant. This underscores a critical nuance: while collaborative engagement is essential, its impact on individual cognition may be mediated by additional factors, such as the quality of group interactions, internalization of shared strategies, or the extent of reflective learning embedded in the experience. Moreover, the relatively small sample sizes in the low- and high-SSCS groups may have constrained the statistical power. Prior studies have shown that collaboration can only enhance individual performance when it is structured to promote metacognitive reflection and the transfer of strategy use from group to individual contexts (Gillies, 2016; O’Donnell et al., 2006) [25, 26]. Table 3 further underscores the value of metacognitive awareness in catalysing learning gains. Although the between-group comparison did not reveal significant differences in post-test performance based on metacognitive level (t(46) = 1.07, *p* = 0.28, Cohen’s d = 0.31, 95% CI [− 2.88, 9.42] for the mean difference), the within-group analysis revealed that students with high baseline metacognitive awareness achieved statistically significant improvements in problem-solving accuracy after the intervention. This reinforces the theories of metacognition as a self-regulatory mechanism that enhances learning through planning, monitoring, and evaluation (Zimmerman, 2002; Flavell, 1979) [27, 13]. These students may have been more adept at engaging with the hands-on and reflective components of the CPSSM module, translating those experiences into measurable improvements. Notably, objective knowledge scores showed a slight numerical decline from pre- to post-intervention in both the moderate (M = 13.96 to 13.24) and high (M = 14.22 to 14.09) metacognitive awareness groups. This contrasts with the gains seen in problem-solving accuracy and may reflect the module’s emphasis on applied, collaborative problem-solving rather than rehearsal of discrete factual knowledge, although regression to the mean or assessment-related factors cannot be excluded. Beyond statistical significance, the overall pre-post gain in problem-solving accuracy (MD = 1.48 on a 50-point scale, corresponding to roughly 3% improvement on this scale) is best described as modest in terms of absolute magnitude. However, in the high-baseline-metacognition subgroup the gain was larger (MD = 2.28, Cohen’s d = 1.53, 95% CI [1.64, 2.92]) and arguably crossed an educationally meaningful threshold, given the short duration of the intervention. Qualitatively, students described shifts in how they approached group tasks, greater role allocation, more explicit planning, and increased confidence in presenting their reasoning to peers and faculty that were not fully captured by the rubric scores. Therefore, we frame the educational meaningfulness of the module as differential: meaningful for learners with stronger baseline metacognitive scaffolding, more modest for the cohort as a whole, and most clearly evident in self-reported changes in collaborative practice rather than in summative scores.

Qualitative reflections captured in Table 5 provided additional insights into students’ learning experiences. Teamwork emerged as the most commonly cited facilitator of success during the model-making task, reaffirming the importance of collaborative learning environments. However, students also noted challenges such as a lack of coordination, limited access to resources, and insufficient domain knowledge, highlighting the logistical and cognitive barriers that can impede group productivity. Interestingly, creativity, although a central intended outcome of the intervention, was rarely cited as a contributing factor, either positively or negatively. This suggests a disconnect between the module’s design intentions and students’ perceived experience, implying a need for clearer scaffolding and assessment of creative thinking within future iterations.

These findings are broadly consistent with previous studies. Brewster et al. (2022) proposed that fostering creativity in healthcare settings involves gathering novel information, being open to disruption, and practicing empathy [5]. While our intervention emphasized these principles, no statistically significant difference in post-test problem-solving was observed across the SSCS-defined creativity levels. Similarly, Ritter and Mostert (2017) found that creative thinking can be trained through structured programs; however, our findings suggest that training must be more explicitly aligned with creativity-oriented outcomes [28]. Urban and Kamila (2024) highlighted that metacognitive and motivational processes play a vital role in creative problem-solving, an insight echoed in our results, where students with higher metacognitive awareness achieved greater gains [7]. Akpur (2025) further emphasized the strong positive correlation between metacognition, critical thinking, and creativity, which may explain why students in our high-awareness group showed higher SSCS scores, reflecting stronger group-level engagement [29]. Moreover, Rivas et al. (2022) demonstrated that educational interventions can strengthen declarative metacognition, which aligns with our observation that students with higher baseline awareness derived greater benefit from the CPSSM module [30]. However, the absence of significant group differences in MCPS scores suggests that while declarative knowledge may improve, applied strategy use may require more immersive or extended intervention.

Finally, the importance of metacognitive monitoring for effective problem-solving has been well documented [31, 32]. Our findings substantiate these claims, showing that learners with higher awareness not only started stronger, but also improved more. Pesout and Nietfeld (2021) and Jaarsveld and Van Leeuwen (2005) also support the idea that creative performance is tied to the precision of metacognitive monitoring, which is likely enhanced through repeated cycles of reflection, planning, and feedback [33, 34]. Collectively, the results of this study indicate that structured educational interventions, such as the CPSSM, can significantly influence metacognitive development, collaborative engagement, and problem-solving efficacy, especially among students already equipped with strong baseline awareness. While collaboration and creativity were central to the module, their translation into individual performance appeared to be dependent on careful instructional design, longitudinal reinforcement, and reflective scaffolding. Future research should include learners from multiple health professions to assess the module’s interprofessional relevance and explore how sustained metacognitive training and explicit creativity prompts can drive deeper, transferable learning outcomes.

We acknowledge that the present module is anchored primarily within the cognitive paradigm of metacognition, problem-solving, and creativity; rather than within the broader holistic framework that includes professional identity formation, empathy, reflective practice, and social responsibility. The three-phase architecture is potentially well-suited for extension into these affective and professional domains in future iterations, particularly through structured reflection prompts within Phase 2 and reflective debriefs following Phase 3. Beyond the measured cognitive outcomes, case-based learning has a recognised potential to foster intrinsic motivation, professional identity formation, reflective practice, empathy, and an appreciation of the human dimensions of medicine. The qualitative emphasis students placed on teamwork and confidence-building suggests that some of these affective gains were experienced, even though they were not formally assessed. An explicit assessment of these dimensions is recommended for future studies.

### Limitations

This study was intentionally conducted within a single professional cohort of first-year medical undergraduates to allow for a focused, context-specific evaluation of the CPSSM module’s effectiveness. While this provided a focused setting to assess improvements in metacognition, collaboration, and problem-solving, the full interprofessional potential of the intervention could not be explored due to the absence of learners from other health disciplines at the time of implementation. In addition, the use of literature-based rather than sample-derived cut-off points for metacognitive awareness grouping produced a very small low-awareness category (*n* = 1), which was excluded from between-group comparisons and limited inference about that subgroup. The four psychometric instruments (MAI, SSCS, MCPS, and GMS) measure related and partially overlapping dimensions of metacognition and self-perception, rather than independent constructs. Several outcomes referenced in the broader discussion such as critical thinking, clinical reasoning, objective creativity, team effectiveness, empathy, and professional identity formation were not directly measured. These should be regarded as conceptual aspirations of the module rather than as measured findings; direct measurement is recommended for future work. The module’s outcome measures are concentrated in the cognitive domain (metacognition, problem-solving, creativity, and collaboration). Affective and professional outcomes, namely empathy, compassion, professional identity formation, reflective capacity, and social responsibility, were not measured in this iteration. Their inclusion is a clear direction for future work, particularly given the early clinical exposure provided by Phase 2 case discussions. Nonetheless, the outcomes lay a strong foundation for future adaptation and scaling of the module in interprofessional contexts. Subsequent research could expand its scope to include nursing, pharmacy, or allied health students to explore the module’s utility in fostering collaborative competencies across diverse professional teams. In addition, the SSCS-based subgroup comparison included very small low (*n* = 5) and high (*n* = 6) groups, limiting the stability and statistical power of this particular analysis. Finally, in-sample Cronbach’s alpha was not separately re-computed for the multi-item scales used here; therefore, reliance on the published reliabilities of the parent instruments is a methodological limitation of the present analysis.

Because the design was single-arm and pre-post, the observed gains could not be attributed to any one phase of the intervention. If a phase-specific contribution must be hypothesised, Phase 1 (integrated teaching) most plausibly underlies the knowledge-linked items, Phase 2 (case-based discussion) the clinical reasoning components, and Phase 3 (collaborative model-making) the applied, creative, and group metacognition outcomes. However, because students experienced all three phases sequentially and were measured only at baseline and end-of-intervention, the present data cannot isolate the contribution of any one phase. Without a parallel control group, part of the pre-post improvement may also reflect testing or learning effects from repeated exposure to the same instruments, maturation across the first-year curriculum, or familiarity with the assessment format, rather than the module itself. Therefore, all reported gains are framed as changes associated with the overall intervention package rather than as causal effects of any one component.

As this study was conducted at a single institution (AIIMS Rajkot) with one cohort of first-year MBBS students, the absolute scores reported here are not directly transferable to other settings. However, the underlying architecture of the module: stratified heterogeneous grouping by baseline metacognition, a three-phase progression from integrated teaching to case-based discussion to collaborative model-making, and multi-disciplinary faculty delivery is specified at a level of granularity intended to be reproducible elsewhere. The module framework, case vignettes, and grading rubric are provided as supplementary materials to support replication. Claims of generalisability will require future multi-site implementation, ideally incorporating a comparison arm or a stepped-wedge design.

## Conclusions

This study suggests that the CPSSM module is an educational approach that may support the development of higher-order cognitive and collaborative skills in undergraduate medical education. The findings support the feasibility and positive reception of the CPSSM module and its association with modest gains in problem-solving accuracy, particularly among learners with higher baseline metacognitive awareness. Stronger claims about generalised educational effectiveness will require controlled, multi-site, longitudinal evaluations before they can be made. Statistically significant differences were observed in problem-solving accuracy across genders, with female students outperforming their male peers in both pre- and post-intervention assessments. Furthermore, students with higher baseline metacognitive awareness showed enhanced collaborative engagement and significant post-intervention improvement in their problem-solving accuracy. These findings suggest that higher baseline metacognitive awareness may be associated with better individual and group learning outcomes. While the application of metacognitive strategies, as measured by the MCPS scale, did not significantly vary across awareness levels, this may reflect the need for prolonged or deeper scaffolding to effectively internalize and deploy such strategies. The CPSSM module’s structure, which integrates multidisciplinary instruction, reflective practice, and creative group tasks, provides a potentially replicable framework designed to support reflective and collaborative competencies. Qualitative data underscored the value of teamwork, while also highlighting barriers such as coordination gaps and limited creativity scaffolding, indicating opportunities for instructional refinement.

### Future directions

The CPSSM module has the potential to support creative problem-solving skills in medical undergraduates over the short term (0–2 years), with potential for horizontal expansion across health disciplines in the intermediate term (3–5 years). While these are plausible long-term aspirations for metacognition-focused curricula, the current single-arm, short-term educational study did not measure clinical or systemic outcomes, and such claims should be tested in future longitudinal, multisite research before being asserted. Nevertheless, despite its promising outcomes, this study is limited by its single-discipline focus, which may affect the generalisability and statistical power of certain findings. Future work should explore the module’s applicability in interprofessional settings, incorporate explicit creativity training, and evaluate the long-term retention and transferability of targeted competencies. By addressing these dimensions, subsequent research can further strengthen the educational, clinical, and systemic relevance of metacognition-driven learning frameworks, such as the CPSSM.

### Key takeaways

Implementation experience suggests four refinements for future iterations: (i) scaffold creativity explicitly in Phase 1, since students rarely cited it spontaneously; (ii) timetable Phase 3 outside assessment-dense weeks and provide model-making materials centrally; (iii) precede Phase 3 with a structured group-process briefing on role allocation and check-ins; and (iv) embed a short metacognitive primer at module start, given the strong baseline metacognition effect on gains.

### Novelty

This study contributes three specific elements to the existing literature: it operationalises metacognition as a stratification variable for composing heterogeneous learning groups (rather than only as an outcome); it integrates a tangible model-making artefact as the externalisation step, providing a single deliverable on which problem-solving, creativity, and collaboration can each be rubric-scored; and it provides a fully specified, reproducible module framework in an Indian first-year MBBS context, where structured metacognitive interventions remain underreported.

## Supplementary Information

Supplementary Material 1.

## Data Availability

The datasets used and/or analysed during the current study are available from the corresponding author on reasonable request.

## Abbreviations

CPSSM: Creative Problem-Solving Skills through Metacognition
CTRI: Clinical Trials Registry–India
GMS: Group Metacognition Scale
ICC: Intraclass correlation coefficient
IEC: Institutional Ethics Committee
KaC: Knowledge about Cognition
MAI: Metacognitive Awareness Inventory
MCPS: Metacognition in Creative Problem-Solving
MCQ: Multiple Choice Questions
MD: Mean Difference
NIRF: National Institutional Ranking Framework
PSA: Problem-Solving Accuracy
RoC: Regulation of Cognition
SD: Standard Deviation
SSCS: Short Scale of Self-Creativity
STEM: Science, Technology, Engineering and Mathematics
TMA: Total Metacognitive Awareness
