# Supplementary material for: Metacognitive team-based learning for creative problem-solving in Indian medical undergraduates: a single-arm pre-post quasi-experimental mixed-methods study
Source: BMC Med Educ. 2026 Aug 15;26:1839. doi: 10.1186/s12909-026-10157-2 (PMC13757174; doi:10.1186/s12909-026-10157-2)
Supplement: Supplementary file 1 [file 12909_2026_10157_MOESM1_ESM.pdf]

# The SQUIRE reporting checklist

For checking that reports that describe systematic work to improve the quality, safety and value of healthcare, using a range of methods to establish the association between observed outcomes and interventions. can be understood and used by everyone

How to use this reporting checklist

This reporting checklist allows authors to demonstrate that their manuscripts adhere to the SQUIRE reporting guideline.

If you have not used a reporting guideline before, read about how and why to use them and check whether SQUIRE is the most applicable reporting guideline for your work.

Reporting guidelines are most useful when used early in research. When writing a manuscript or application, consider using the full guidance where you'll find explanations and examples for each item.

After writing, demonstrate adherence by completing this checklist:

1. Specify where each item is described (see Note 1).
2. Cite this checklist (See Note 2).
3. Include your completed checklist as a supplement when submitting to a journal so that future readers can use it to find information.

| | Item Description | Location (or reason for not reporting) |
|---|---|---|
| **Title and Abstract** | | |
| 1. Title | Indicate that the manuscript concerns an initiative to improve healthcare (broadly defined to include the quality, safety, effectiveness, patient-centeredness, timeliness, cost, efficiency and equity of healthcare). | Yes. Page 1, Lines 1–3. |
| | **EDU 1:** Indicate that the manuscript concerns efforts to improve health professions education systems and learning. | Yes. The title explicitly names the educational module (CPSSM) and its evaluation. Page 1, Lines 1–3. |
| 2. Abstract | 2a. Provide adequate information to aid in searching and indexing.<br><br>2b. Summarise all key information from various sections of the text using the abstract format of the intended publication or a structured summary such | Yes, structured format present (Background, Methods, Results, Conclusions, Trial registration). Submitted as a separate Abstract file accompanying this manuscript, per the journal's |

| | | |
|---|---|---|
| | as: background, local problem, methods, interventions, results, conclusions. | submission system; not part of the paginated main-text document. |
| | **EDU 2:** Keywords include a focus on education and learning. | Yes. Keywords focus on education/learning (Metacognition; Creative problem-solving; Medical education; Team-based learning; Undergraduate medical students; Mixed-methods; Anatomy education). Submitted with the separate Abstract file. |
| **Introduction** | | |
| 3 & 4. Problem description & Available Knowledge | 3. Nature and significance of the local problem. | Yes. Introduction, Paragraph 1, Page 1, Lines 15–33. |
| | 4. Summary of what is currently known about the problem, including relevant previous studies. | Yes. Introduction, Paragraph 1, Page 1 Line 19 – Page 2 Line 33 (citations 1–7, including NIRF 2021 data). |
| | **EDU 3:** Description of the nature and significance of the need for change in the local educational system. | Yes. Introduction, Paragraphs 1 & 4, Page 1 Lines 15–33 and Page 2 Line 50 – Page 3 Line 65. |
| 5. Rationale | Informal or formal frameworks, models, concepts, and/or theories used to explain the problem, any reasons or assumptions that were used to develop the intervention(s), and reasons why the intervention(s) was expected to work. | Yes. Introduction, Paragraph 2, Page 2, Lines 34–41 (role of metacognition in creative problem-solving). |
| | **EDU 5:** Identify the guiding theory (learning, change, implementation, or other) and how it aligns with the need for change in the local educational system. | Yes. Introduction, Paragraphs 2 & 5, Page 2 Lines 34–41 and Page 3 Lines 66–73 (metacognitive theory and rationale for a multi-disciplinary, faculty-led design). |
| 6. Specific aims | Purpose of the project and of this report. | Yes. Introduction, Paragraph 3, Page 2, Lines 42–49 (three a priori objectives, research question, and pre-specified primary outcome). |
| **Methods** | | |
| 7. Context | Contextual elements considered important at the outset of introducing the intervention(s). | Yes. Methods, Section 2.1 ‘Study Design and Educational |

| | | |
|---|---|---|
| | | Setting', Page 3 Line 75 – Page 4 Line 81. |
| | **EDU 7a:** Contextual elements for learning (e.g. setting, program, people, resources, social, geopolitical influences) before the intervention(s). | Yes. Methods, Sections 2.1 and 2.3 'Participants and Sampling', Page 3 Line 75 – Page 4 Line 81, and Page 4 Lines 98–104. |
| | **EDU 7b:** The interrelationships between the contextual elements and the local educational and healthcare systems before the intervention(s). | Yes. Methods, Section 2.1, Page 3 Lines 76–81 (AIIMS Rajkot as an Institute of National Importance; Department of Anatomy led the module with cross-disciplinary faculty involvement). |
| 8. Intervention(s) | A. Description of the intervention(s) in sufficient detail that others could reproduce it | Yes. Methods, Section 2.5 'Intervention: CPSSM Module Structure', Page 4 Line 107 – Page 6 Line 171. |
| | B. Specifics of the team involved in the work. | Yes. Methods, Section 2.5, Page 5, Lines 133–137 (roles of the Principal Investigator, clinician faculty, psychiatrist, and full interdisciplinary faculty team). |
| | **EDU 8a:** Description of the primary intervention(s) and co-interventions (e.g. faculty or tool development). | Yes. Methods, Section 2.5, Page 4 Line 107 – Page 6 Line 171 (three-phase module structure, case design, and faculty/tool development). |
| | **EDU 8b:** Specify how the interprofessional education team (e.g. faculty staff, patients, and learners) was part of the design of the intervention. | Yes. Introduction, Page 3 Lines 66–73, and Methods Section 2.5, Page 4 Lines 107–109 and Page 5 Lines 133–137 (multi-disciplinary faculty team's role in designing and delivering the module). |
| 9. Study of the Intervention(s) | A. Approach chosen for assessing the impact of the intervention(s) | Yes. Methods, Section 2.7 'Data Analysis', Page 8 Lines 219–227. |
| | B. Approach used to establish whether the observed outcomes were due to the intervention(s). | Yes. Methods, Section 2.1, Page 3 Lines 88–92, and Section 2.7, Page 8 Lines 223–227 (single-arm design; pre-post changes reported as |

| | | |
|---|---|---|
| | | associations, not causal effects). |
| | **EDU 9a:** Approach used to understand the impact of the educational intervention(s) on the learner and beyond, such as impact on patients, families, the community, faculty, educational programs, or the healthcare system. | Yes. Methods, Sections 2.6.2 and 2.7, Page 7 Line 206 – Page 8 Line 210, and Page 8 Line 229 – Page 9 Line 236 (module feedback questionnaire and thematic analysis of learner-reported facilitators/barriers). |
| | **EDU 9b:** Approach to assess the fidelity of and the iterative changes to the planned intervention(s) over time. | Yes. Methods, Section 2.5, Page 5 Lines 137–142 (identical time structure maintained across all ten case modules) and Lines 166–169 (inter-rater reliability of model-making scores); Section 2.7, Page 8 Line 232 – Page 9 Line 236 (inter-coder reliability for qualitative coding). |
| 10. Measures | A. Measures chosen for studying processes and outcomes of the intervention(s), including rationale for choosing them, their operational definitions, and their validity and reliability | Yes. Methods, Sections 2.6.1 'Pre-Intervention Assessments' and 2.6.2 'Post-Intervention Assessments', Page 6 Line 174 – Page 8 Line 210; reliability coefficients reported in Section 2.7, Page 9 Lines 237–240. NB: Page 4, Line 102 also lists RIPLS and IEPS among questionnaires collected, but neither appears in Table 1, this Methods section, or the Results — RIPLS and IEPS were administered as part of the broader registered project but are not reported in this manuscript; this is disclosed in Supplementary File 7. |
| | B. Description of the approach to the ongoing assessment of contextual elements that contributed to t… | Yes. Methods, Section 2.6.3 'Data Collection Logistics', Page 8, Lines 212–218. |

| | | |
|---|---|---|
| | **EDU 10:** Quantitative and/or qualitative measures chosen to assess the educational processes and outcomes on learners, faculty, educational programs, patients, families, healthcare systems, or communities. | Yes. Methods, Sections 2.6.1 and 2.6.2, Page 6 Line 174 – Page 8 Line 210 (MAI, MCQ, problem-solving questionnaire, SSCS, model-grading rubric, MCPS, GMS, and module feedback questionnaire). NB: RIPLS/IEPS appear in Page 4 Line 102's instrument list but not here — unresolved inconsistency flagged for the author. |
| 11. Analysis | A. Qualitative and quantitative methods used to draw inferences from the data | Yes. Methods, Section 2.7 'Data Analysis', Page 8 Line 219 – Page 9 Line 236 (descriptive/inferential statistics and Braun & Clarke thematic analysis). |
| | B. Methods for understanding variation within the data, including the effects of time as a variable. | Yes. Methods, Section 2.7, Page 8 Lines 221–227 (paired pre–post comparisons) and Page 9 Lines 251–255 (effect sizes, 95% CIs, and normality/variance checks with non-parametric alternatives where assumptions were violated). |
| 12. Ethical considerations | Ethical aspects of implementing and studying the intervention(s) and how they were addressed, including, but not limited to, formal ethics review and potential conflict(s) of interest. | Yes. Methods, Section 2.2 'Ethical Approval and Trial Registration', Page 4, Lines 93–97; Declarations 7.1 'Ethics approval and consent to participate' and 7.5 'Competing interests', Page 20, Lines 564–568 and Line 578. |
| | **EDU 12:** Approaches to address vulnerability of learner participants | Yes. Methods, Section 2.3 'Participants and Sampling', Page 4, Lines 99–100 (written informed consent obtained from each student); Declarations 7.1, Page 20, Lines 564–568. |
| **Results** | | |

| | | |
|---|---|---|
| 13 a & b. Evolution of the intervention and details of process measures | 13a. Initial steps of the intervention(s) and their evolution over time (e.g., time-line diagram, flow chart, or table), including modifications made to the intervention during the project. | Not applicable. The CPSSM module was implemented once, with a fixed structure across all ten case topics (Methods, Section 2.5, Page 4 Line 107 – Page 6 Line 171); no within-study modification occurred. Proposed refinements for future iterations are reported under 'Key Takeaways', Page 19, Lines 535–540. |
| | 13b. Details of the process measures and outcome. | Yes. Results, Sections 3.1.1–3.1.4, Page 10 Line 275 – Page 11 Line 315 (Tables 2–4, Figure 2), and Section 3.1.5, Page 11 Line 316 – Page 12 Line 349 (Table 5, Figure 3). |
| | **EDU 13:** For each educational intervention and co-intervention, provide details about iterative modifications based on the assessment of the learning. | Not applicable. No iterative, within-study modification of the intervention was undertaken (single fixed-design implementation). Suggested modifications for future iterations are reported under 'Key Takeaways', Page 19, Lines 535–540. |
| 13 c, d & e Contextual elements and unexpected consequences | 13c. Contextual elements that interacted with the interventions | Yes. Results, Sections 3.1.2–3.1.4, Page 10 Line 291 – Page 11 Line 315 (baseline metacognitive level as a contextual/stratifying factor), and Discussion, Page 13 Line 377 – Page 14 Line 387. |
| | 13d. Observed associations between outcomes, interventions and relevant contextual factors | Yes. Results, Section 3.1, Page 10 Line 270 – Page 12 Line 349, and Discussion, Page 13 Line 363 – Page 16 Line 464. |
| | 13e. Unintended consequences such as benefits, harms, unexpected results, problems or failures associated with the intervention(s) | Yes. Discussion, Page 14 Line 407 – Page 15 Line 409 (unexpected numerical decline in objective knowledge scores from pre- to post-intervention in the moderate- and high-metacognition groups, contrasting with the gains |

| | | |
|---|---|---|
| | | observed in problem-solving accuracy). |
| 13 e. Missing data | 13e. Details about missing data. | Partially applicable. Methods, Section 2.3 'Participants and Sampling', Page 4, Lines 99–101 (1 of 50 enrolled students remained absent throughout the intervention and was excluded, giving a final analytic sample of 49; no other missing data were reported). |
| **Discussion** | | |
| 14. Summary | 14a. Key findings, including relevance to the rationale and specific aims | Yes. Discussion, Page 13 Line 363 – Page 14 Line 376, and 'Summary of Results', Page 12 Line 351 – Page 13 Line 361. |
| | 14b. Particular strengths of the project | Yes. 'Novelty', Page 19, Lines 541–546. |
| | **EDU 14:** Connect the findings to the guiding theory (learning, change, implementation, other) used to direct the change in the local educational system. | Yes. Discussion, Page 14 Line 398 – Page 16 Line 464 (findings linked to metacognitive self-regulation theory, citing Zimmerman 2002 and Flavell 1979). |
| 15. Interpretation | 15a. Nature of the association between the intervention(s) and the outcomes | Yes. 'Summary of Results', Page 12 Line 358 – Page 13 Line 361, and Discussion, Page 13 Line 363 – Page 15 Line 422 (associations explicitly framed as non-causal given the single-arm design). |
| | 15b. Comparison of results with findings from other publications | Yes. Discussion, Page 15 Line 433 – Page 16 Line 464 (compared with Brewster et al., Ritter & Mostert, Urban & Urban, Akpur, Rivas et al., Dulger & Ogan-Bekiroglu, Cohors-Fresenborg et al., Pesout & Nietfeld, and Jaarsveld & Van Leeuwen). |
| | 15c. Impact of the project on people and systems | Reported as an aspirational/future impact rather than a measured one. |

| | | |
|---|---|---|
| | | Discussion, Page 16 Line 465 – Page 17 Line 476, and Limitations, Page 17 Lines 487–492. |
| | 15d. Reasons for any differences between observed and anticipated outcomes, including the influence of context | Yes. Discussion, Page 14 Lines 384–387 (need for sustained engagement) and Lines 407–408 (explanation for the unexpected knowledge-score decline); Page 14 Lines 388–397 (why higher SSCS scores did not translate into individual performance gains). |
| | 15e. Costs and strategic trade-offs, including opportunity costs | Not applicable. No cost or resource-utilisation data were collected or analysed in this study. |
| | **EDU 15:** Include the impact of the intervention(s) on learners, faculty, educational program, patients, families, healthcare systems, or communities. | Reported as a future/aspirational impact, not a measured outcome (explicitly flagged as unmeasured in Limitations, Page 17 Lines 487–492). Discussion, Page 16 Line 465 – Page 17 Line 476. |
| 16. Limitations | 16a. Limits to the generalizability of the work. | Yes. Limitations, Page 17, Lines 478–482 (single professional cohort, single institution) and Page 18, Lines 500–502 (small SSCS subgroup sizes, n=5 and n=6). |
| | 16b. Factors that might have limited internal validity such as confounding, bias, or imprecision in the design, methods, measurement, or analysis. | Yes. Limitations, Page 17 Lines 486–492 (overlapping psychometric constructs; several discussed outcomes, e.g. critical thinking, clinical reasoning, empathy, were not directly measured) and Page 18 Lines 503–504 (in-sample Cronbach's alpha not recomputed). |
| | 16c. Efforts made to minimize and adjust for limitations. | Yes. Methods, Section 2.5, Page 6 Lines 166–169 (blinded independent rating of models; inter-rater ICC = 0.88) and Section 2.7, Page 8 Line 232 – Page 9 Line 236 (independent |

| | | |
|---|---|---|
| | | dual-coding of qualitative data to consensus; Cohen's κ = 0.87) and Page 9 Lines 251–255 (assumption checks with non-parametric alternatives used where needed). |
| 17. Conclusion | 17a. Usefulness of the work<br>17b. Sustainability<br>17c. Potential for spread to other contexts | Yes. Conclusions, Page 18, Lines 505–522, and 'Future directions', Page 18 Line 523 – Page 19 Line 534. |
| | 17d. Implications for practice and for further study in the field<br>17e. Suggested next steps | Yes. 'Future directions', Page 18 Line 523 – Page 19 Line 534, and 'Key Takeaways', Page 19, Lines 535–540. |
| | **EDU 17b:** Scalability of the work to other learners and contexts.<br>- - | Yes. Conclusions, Page 18, Lines 518–520 ('replicable and scalable approach'); 'Future directions', Page 19 Lines 529–531; and Limitations, Page 18 Lines 496–499 (proposed expansion to nursing, pharmacy, and allied health learners). |
| | **EDU 17d:** Lessons learned for clinical practice, education and policy. | Yes. 'Key Takeaways', Page 19, Lines 535–540, and 'Novelty', Page 19, Lines 541–546. |
| **Other information** | | |
| 18. Funding | Sources of funding that supported this work. Role, if any, of the funding organization in the design, implementation, interpretation and reporting. | Yes. Declarations, Section 7.6 'Funding', Page 20, Lines 579–581. |
| | | Yes. Declarations, Section 7.6 'Funding', Page 20, Lines 579–581 (no role of the funding body in study design, implementation, interpretation, or reporting beyond financial support). |

## 1 How to specify where content is

Tell the reader where they can find information. E.g.,

- Results; paragraph 2
- Methods, Participants; paragraphs 1 & 2.
- Table 3
- Supplement B, para. 4

If you have chosen not to describe an item, explain why. You can do this in the checklist, or as a note below it.

You can describe items in the article body, or in tables, figures, or supplementary materials, and should prioritize items you feel are most important to your intended audience. The order of items in your manuscript does not need to match the order of items in this checklist. You can decide how best to structure your work.

## 2 How to cite

Describe how you used SQUIRE at the end of your Methods section, referencing the resources you used e.g.,

> 'We used the SQUIRE reporting guideline(1) to draft this manuscript, and the SQUIRE reporting checklist(2) when editing, included in supplement A'

If you use a reporting checklist, remember to include it as a supplement when publishing so that readers can easily find information and see how you have interpreted the guidance.

1. Ogrinc G, Davies L, Goodman D, Batalden P, Davidoff F, Stevens D. SQUIRE 2.0 (standards for QUality improvement reporting excellence): Revised publication guidelines from a detailed consensus process. BMJ Quality & Safety [Internet]. 2016 Dec;25(12):986–92. Available from: https://qualitysafety.bmj.com/content/25/12/986

2. Ogrinc G, Davies L, Goodman D, Batalden P, Davidoff F, Stevens D. The SQUIRE reporting checklist. In: Harwood J, Albury C, Beyer J de, Schlüssel M, Collins G, editors. The EQUATOR network reporting guideline platform [Internet]. The UK EQUATOR Centre; 2025. Available from: https:/resources.equator-network.org/reporting-guidelines/squire/squire-checklist.docx

**IEC Approval Number: AIIMS/RAJKOT/6th IEC/FB/47** **Date: 04/02/2025**

# APPROVAL LETTER

To,
Dr. Sundip Charmode,

Department of Anatomy,

AIIMS, Rajkot.

**Protocol Title: Enhancing creative problem-solving skills among first year medical students using a Metacognition module and team-based approaches.**

The meeting of the Institutional Ethics Committee (IEC) AIIMS, Rajkot was held at AIIMS Rajkot with Dr. Gauravi Dhruva as Chairperson. The list of members who attended the meeting is as follows.

| |
|---|
| Dr. Gauravi Dhurva, Chairperson |
| Dr. (Col) Ashwini Agarwal, Member, Dean Research |
| Dr. Simmi Mehra, Member |
| Dr. Ragini Singh Member; Basic Medical Scientist |
| Dr. K. K Rawal |
| Dr. Krupal Joshi, Member; Epidemiologist |
| Dr. Sachin Singh, Member; Clinician |
| Dr. Shubha Singhal, Member; Pharmacologist |
| Mr. Himanshu Mankad, Member; Social Scientist (External) |
| Mr. Vinay Jasani, Member; Lay Person (External) |
| Dr. Abhishek Padhi, Member Secretary |

It is hereby confirmed that neither you nor any of the study team members have participated in the voting/decision making procedures of the committee.

The IEC has reviewed and approved the following documents submitted for the above – mentioned clinical study.

Institutional Ethics Committee All India Institute of Medical Sciences IEC RAJKOT

1. Study Protocol
2. Informed Consent form
3. Participant Information Sheet

Outward No./ AIIMS Rajkot
Institutional Ethics Committee/ 2025 / 55
Date : 06/02/2025

Inward No./ AIIMS Rajkot
Anatomy Department/ 02 / 13
Date : 06/02/25



Scanned with CamScanner

The IEC hereby approves the proposal entitled: **Enhancing creative problem-solving skills among first year medical students using a Metacognition module and team-based approaches.**

This approval is valid till for the entire duration of the study. It is the policy of IEC that it be informed about any serious adverse event occurring during the course of the study within seven working days of the occurrence of the adverse event. If 'Death' is a SAE, it should be reported to the IEC within 24 hours of its occurrence via an e-mail.

No deviations from, or changes of the protocol and Informed Consent Document should be initiated without prior written approval by the IEC of an appropriate amendment. The IEC expects that the investigator should promptly report to the IEC any deviations from, or changes of, the protocol to eliminate immediate hazards to the trial subjects and about any new information that may affect adversely the safety of the subjects or the conduct of the trial.

For studies which will continue for more than a year, a continuing review report needs to be submitted 6 months. A copy of the final report should be submitted to the IEC for review.

**The recruitment of study subjects can be started only after obtaining permission from CTRI/DCGI/any other regulatory bodies as applicable.**

Institutional Ethics Committee · IEC RAJKOT · All India Institute of Medical Sciences

Prof. Dr. Gauravi Dhruva
Chairperson
IEC, AIIMS Rajkot

अध्यक्ष / CHAIRMAN
संस्थागत आचार समिति
INSTITUTIONAL ETHICS COMMITTEE
अखिल भारतीय आयुर्विज्ञान संस्थान, राजकोट
ALL INDIA INSTITUTE OF MEDICAL SCIENCES, RAJKOT, GUJARAT

**Date of approval of the study: 04/02/2025**



Scanned with CamScanner

## Clinical Trial Details

| | |
|---|---|
| **CTRI Number** | CTRI/2025/02/080559 [Registered on: 14/02/2025] - **Trial Registered Prospectively** |
| **Last Modified On** | 09/07/2025 |
| **Post Graduate Thesis** | No |
| **Type of Trial** | Observational |
| **Type of Study** | Quasi experimental study using mixed method analysis |
| **Study Design** | Single Arm Study |
| **Public Title of Study** | Helping first-year medical students to think creatively and solve problems better using teamwork |
| **Scientific Title of Study** | Enhancing creative problem-solving skills among first year medical students using a Metacognition module and team-based approaches. |

**Secondary IDs if Any**

| Secondary ID | Identifier |
|---|---|
| NIL | NIL |

**Details of Principal Investigator**

| Details of Principal Investigator | |
|---|---|
| **Name** | Dr Sundip Charmode |
| **Designation** | Associate Professor |
| **Affiliation** | All-India Institute of Medical Sciences Rajkot |
| **Address** | Department of Anatomy, Ground floor, Academic block, AIIMS Rajkot, KHANDHERI, RAJKOT-360110<br>Rajkot<br>GUJARAT<br>360110<br>India |
| **Phone** | 09633239685 |
| **Fax** | |
| **Email** | sundip.charmode@yahoo.com |

**Details Contact Person (Scientific Query)**

| Details Contact Person (Scientific Query) | |
|---|---|
| **Name** | Dr Sundip Charmode |
| **Designation** | Associate Professor |
| **Affiliation** | All-India Institute of Medical Sciences Rajkot |
| **Address** | Department of Anatomy, Ground floor, Academic block, AIIMS Rajkot, KHANDHERI, RAJKOT-360110<br>Rajkot<br>GUJARAT<br>360110<br>India |
| **Phone** | 09633239685 |
| **Fax** | |
| **Email** | sundip.charmode@yahoo.com |

**Details Contact Person (Public Query)**

| Details Contact Person (Public Query) | |
|---|---|
| **Name** | Dr Sundip Charmode |

| | |
|---|---|
| **Designation** | Associate Professor |
| **Affiliation** | All-India Institute of Medical Sciences Rajkot |
| **Address** | Department of Anatomy, Ground floor, Academic block, AIIMS Rajkot, KHANDHERI, RAJKOT-360110<br>Rajkot<br>GUJARAT<br>360110<br>India |
| **Phone** | 09633239685 |
| **Fax** | |
| **Email** | sundip.charmode@yahoo.com |

**Source of Monetary or Material Support**

| Source |
|---|
| Department of Anatomy, All India Institute of Medical Sciences (AIIMS) Rajkot, Gujarat-360110 |

**Primary Sponsor**

| Primary Sponsor Details | |
|---|---|
| **Name** | Dr Sundip Charmode |
| **Address** | Department of Anatomy, Academic block, AIIMS Rajkot, KHANDHERI, RAJKOT-360110 |
| **Type of Sponsor** | Other [All the expenses will be paid by the principal investigator from his Learning resources allowance. ] |

**Details of Secondary Sponsor**

| Name | Address |
|---|---|
| NIL | NIL |

**Countries of Recruitment**

| List of Countries |
|---|
| India |

**Sites of Study**

| Principal Investigator | Site Name | Address | Contact |
|---|---|---|---|
| Dr Sundip Charmode | All India Institute of Medical Sciences (AIIMS) Rajkot | Department of Anatomy, Academic block, AIIMS Rajkot, KHANDHERI, RAJKOT-360110<br>Rajkot, GUJARAT | 09633239685 |

**Details of Ethics Committee**

| Name | Approval Status |
|---|---|
| Institutional Ethical committee, AIIMS Rajkot | Approved |

**Regulatory Clearance Status from DCGI**

| Status |
|---|
| Not Applicable |

**Health Condition / Problems Studied**

| Health Type | Condition |
|---|---|
| Healthy Human Volunteers | Normal healthy human volunteers shall be selected. First year MBBS undergraduate students will be selected. |

**Intervention / Comparator Agent**

| Type | Name | Details |
|---|---|---|
| Intervention | NIL | NIL |
| Comparator Agent | NIL | NIL |

**Inclusion Criteria**

| Inclusion Criteria | |
|---|---|
| **Age From** | 16.00 Year(s) |
| **Age To** | 75.00 Year(s) |
| **Gender** | Both |

| | |
|---|---|
| **Details** | 1st MBBS medical undergraduates who will give informed consent. <br/> |

**Exclusion Criteria**

| Exclusion Criteria | |
|---|---|
| **Details** | 1st MBBS medical undergraduates who will not give informed consent. |

**Method of Generating Random Sequence**

Coin toss, Lottery, toss of dice, shuffling cards etc

**Method of Concealment**

An Open list of random numbers

**Blinding/Masking**

Participant and Investigator Blinded

**Primary Outcome**

| Outcome | Timepoints |
|---|---|
| 1.Short term outcome (0-1 years):<br>a. Acquiring enhanced creative problem-solving skills amongst medical undergraduate students in their 1st professional year.<br>b.Better retention of creative problem-solving skills amongst medical undergraduate students in their 2nd professional year. | Milestone Targets Date<br>Acquiring the copyright permission for using the 52-items MAI scale, Collecting all the evaluation items, Standardizing and Validating them to construct a new metacognition measurement scale for the project. By the end of 2nd month after ethical approval. 31st December 2024<br>Execution of the module in the institution amongst the 1st year medical undergraduate students. By the end of 6th month after ethical approval. 31st May 2025<br>Qualitative and quantitative data collection, its evaluation, Compilation of data, master chart preparation, statistical analysis and final report preparation By the end of 8th months 31st July 2025<br>Publication of study findings. By the end of 12 months. 31st August 2025 |

**Secondary Outcome**

| Outcome | Timepoints |
|---|---|
| 2. Intermediate outcome (3-5 years):<br>a. Acquiring enhanced creative problem-solving skills amongst medical professionals.<br>b. Acquiring enhanced creative problem-solving skills amongst the non-medical professionals.<br><br><br>3. Long term outcome (5-7 years):<br>a. Reduced misdiagnoses and complications in the patients reporting to the institute.<br>b. Enhanced the overall patient health outcomes.<br>c. Enhanced patient safety and satisfaction.<br>d. Reduced financial burden on the patient and his/her family members. | Intermediate outcome (3-5 years):<br>Milestones<br>a. Acquiring enhanced creative problem-solving skills amongst medical professionals.<br><br>Time period<br>3rd and 4th year after the project gets completed.<br><br>Milestones<br>b. Acquiring enhanced creative problem-solving skills amongst the non-medical professionals.<br><br>Time period<br>4th and 5th year after the project gets completed.<br><br><br>3. Long term outcome (5-7 years):<br>Milestones<br>a. Reduced misdiagnoses and complications in the patients reporting to the institute.<br>b. Enhanced the overall patient health outcomes.<br>c. Enhanced patient safety and satisfaction.<br>d. Reduced financial burden on the patient and his/her family members.<br><br>Time period<br>Between 5th and 7th year after the project gets |

| | |
|---|---|
| | completed, all these four long-term outcomes shall be acquired. |
| **Target Sample Size** | Total Sample Size: **50**<br>Sample Size from India: **50**<br>Final Enrollment (Total): **49**<br>Final Enrollment (India): **49** |
| **Phase of Trial** | N/A |
| **Date of First Enrollment (India)** | 01/03/2026 |
| **Date of First Enrollment (Global)** | No Date Specified |
| **Date of Study Completion (India)** | No Date Specified |
| **Date of Study Completion (Global)** | No Date Specified |
| **Estimated Duration of Trial** | Years: 1, Months: 0, Days: 0 |
| **Recruitment Status (Global)** | Not Applicable |
| **Recruitment Status (India)** | Completed |
| **Publication Details** | N/A |
| **Brief Summary** | Design: Quasi experimental study using mixed-method analysis<br><br>Settings: Department of Anatomy of AIIMS Rajkot<br><br>Sampling technique: Universal Sampling method.<br><br>Sample Size Calculation: Universal sampling method as all the available 50 first year medical students will be included after taking their informed consent.<br><br>Sample size (n) = Universal sampling of 1st year medical students.<br><br>Eligibility Criteria<br><br>Inclusion:<br><br>1. 1st MBBS students who will give informed consent.<br><br>Exclusion:<br><br>1. 1st MBBS students who will not give informed consent.<br><br>Data collection procedure: The study participants will be assessed before and after the module implementation. Pre-test before module implementation shall include assessment of metacognition awareness using a self-designed questionnaire which will be prepared from the currently available 'Metacognition Awareness Inventory' (MAI) 52-items scale in public domain. Copyright permission to use this scale for designing our scale has been successfully acquired. Additionally, an objective assessment (Pre-test) for assessing the domain specific for knowledge on the case will be conducted. Apart from these mentioned assessments, evaluation regarding the Readiness for Interprofessional Learning Scale using the (RIPLS) scale, creativity of the students using the 'Short scale for creative self-efficacy' (SSCS) and 26 items, Creative Self-Regulation (CSR) scale shall be done provided the copyright for the same is acquired and time feasibility. After the module implementation, Model Scoring Scale' specifically designed for this project shall be used to assess the functional (working) models prepared by students for its creativity, problem-solving accuracy, followed by collaborative skills, and teamwork of the students. In addition, an objective assessment |

(Post-test) followed by module feedback from students and faculty and IP team shall be collected. The 'Module Feedback Questionnaire' and 'Model Grading Scale' have been specially designed for the project by the principal and co-investigators.

**Informed Consent Form**

Study Title: Enhancing creative problem-solving skills among first year medical students using a Metacognition module and team-based approaches.

Study Number:

Subject's Initials: ________________________ Subject's Name: _____________________

Date of Birth/Age: _________

Address of the Subject: ___

Qualification: _____________

Occupation: Student or Self-Employed or Service or Housewife or Others (Please click as appropriate)

Annual Income of the subject:

Name and address of the nominees and his relation to the subject (for the purpose of compensation in case of trial related death)

i) I confirm that I have read and understood the information sheet dated ___________ for the above study and have had the opportunity to ask questions.

(ii) I understand that my participation in the study is voluntary and that I am free to withdraw at any time, without giving any reason, without my medical care or legal rights being affected.

(iii) I understand that the Sponsor of the clinical trial, others working on the Sponsor's behalf, the Ethics Committee and the regulatory authorities will not need my permission to look at my health records both in respect of the current study and any further research that may be conducted in relation to it, even if I withdraw from the trial.

iv) I agree to this access. However, I understand that my identity will not be revealed in any information released to third parties or published.

(v) I agree not to restrict the use of any data or results that arise from this study provided such a use is only for scientific purposes.

(vi) I agree to take part in the above study.

Signature (or Thumb impression) of the Subject/Legally Acceptable Representative/Legal guardian:

Signatory's Name: ___________________________________________________

Signature of the Investigator: _____________ Date: ____ / ____ /

Study Investigator's Name: ___ ___________________

Signature of the Witness ______________________________ Date: ____ / ____ /

Name of the Witness: ________________________

Copy of the Patient Information Sheet and duly filled Informed Consent Form shall be handed over to the subject his or her attendant.

**Supplementary file 5**

**CPSSM Module Framework: Ten Case-Based Anatomy Modules Integrating Metacognition Training, Creative Problem-Solving, and Collaborative Skills Assessment**

**Author:** Dr. Sundip Charmode, Dr. Simmi Mehra, Prof. Ciraj Ali Mohammed, Prof. Abdul Ajees Abdul Salam

*This file contains the detailed framework for all ten case-scenario modules of the Creative Problem-Solving Skills Module (CPSSM), an integrated teaching-learning and assessment activity for first-year medical students. Each module follows a consistent three-step structure (integrated lecture-theatre session, clinical case-based discussion, and a group model-making activity in the dissection hall) and is assessed using a pre-test/post-test design incorporating metacognitive awareness, creative problem-solving, model-grading, and feedback instruments. References cited within this file are listed in a single, consolidated reference list at the end and are not repeated in the main manuscript reference list.*

## Module 1: Liver

**Aim:** The learner should be able to describe the anatomical position, presenting parts, relations, lobes (anatomical and physiological), hepatic segments, hepatic and portal circulation, and applied anatomy of the liver.

**Objectives:**

- To enhance the problem-solving skills of the learners required for understanding the anatomical basis of common liver pathologies.
- To enhance collaborative and creative skills necessary for effective participation in a group activity like 'model making task'.

**Pre-test:**

- Assessment of metacognitive awareness using the Metacognition Awareness Inventory (MAI), a 52-item scale. [Dr. Sundip Charmode]
- Objective assessment of students' baseline knowledge of the anatomical position, presenting parts, relations, lobes, hepatic segments, porto-systemic circulation, and applied anatomy of liver. [Dr. Sundip Charmode]

**Module contents:** The module consists of 3 steps.

- Step 1 and Step 2 will be conducted as an integrated session in the lecture theatre.
- Step 3 will be a group activity (model-making task) conducted in the dissection hall.

*Step 1 and 2: Integrated session (55 minutes)*

| Step | Topic / session | Duration | Department | Faculty |
|---|---|---|---|---|
| Step 1 | Gross anatomy of liver | 20 minutes | Anatomy | Dr. Sundip Charmode |
| | Applied anatomy of liver | 15 minutes | Medicine / General Surgery | Medicine / General Surgery |
| | Application of metacognition skills and strategies | 10 minutes | Anatomy | Dr. Sundip Charmode |
| Step 2 | Clinical case-based discussion | 10 minutes | Anatomy | Dr. Sundip Charmode |

**Step 2 – Case scenario:** A 52-year-old male with a history of chronic alcohol use presents with abdominal swelling, vomiting blood, and fatigue. Examination shows ascites, splenomegaly, and oesophageal varices. Investigations confirm portal vein dilation. What is the likely diagnosis and the possible differential diagnosis in this patient? Mention the investigations appropriate for diagnosing this condition. Also discuss the various complications in case this condition is left untreated.

**Step 3 – Group activity:** Roll numbers 1–5 [Dissection hall]. Structural model making on '***Liver showing the porto-systemic anastomoses and portal hypertension***'.

**Post-test:**

- Models will be evaluated using the Model Grading Scale and rated on creativity, communication skills, and problem-solving accuracy. [Dr. Sundip Charmode / other Anatomy faculty]
- Application of metacognitive skills in problem-solving tasks will be assessed using a self-designed Creative Problem-Solving Scale. [Dr. Sundip Charmode]
- Objective assessment (post-test). [Dr. Sundip Charmode]
- Feedback will be collected from students and faculty (Anatomy) using a Module Feedback Questionnaire. [Dr. Sundip Charmode]

**Instructions for the group activity (model-making task):**

- Roles and responsibilities: All group members should assign distinct roles and responsibilities to each other for the task.
- Materials: Use eco-friendly materials, such as clay, cardboard, or colored wires for blood vessels, bare areas, lobes, hepatic segments and related structures.
- Anatomical accuracy: Label important sites of porto-systemic anastomoses and indicate the flow of blood in portal hypertension.
- Creativity: Use color codes and creative elements to distinguish portal and systemic circulation.
- Submit by the deadline given; a presentation will be conducted on the date specified.

**Aim:** The learner should be able to describe the anatomical location, extent, boundaries, contents, defense mechanism, and applied anatomy of the inguinal canal.

**Objectives:**

- To enhance problem-solving skills required to understand the anatomical basis of all the types of inguinal hernias.
- To enhance collaborative and creative skills necessary for effective participation in a group activity like 'model making task'.

**Pre-test:**

- Assessment of metacognitive awareness using the Metacognition Awareness Inventory (MAI), a 52-item scale. [Dr. Sundip Charmode]
- Objective assessment of students' baseline knowledge of the anatomical position, extent, boundaries, contents, defense mechanism, and applied anatomy of the inguinal canal. [Dr. Sundip Charmode]

**Module contents:** The module consists of 3 steps.

- Step 1 and Step 2 will be conducted as an integrated session in the lecture theatre.
- Step 3 will be a group activity (model-making task) conducted in the dissection hall.

*Step 1 and 2: Integrated session (55 minutes)*

| Step | Topic / session | Duration | Department | Faculty |
|---|---|---|---|---|
| Step 1 | Gross anatomy of inguinal canal | 20 minutes | Anatomy | Dr. Sundip Charmode |
| | Applied anatomy of inguinal canal | 15 minutes | General Surgery | General Surgery |
| | Application of metacognition skills and strategies | 10 minutes | Anatomy | Dr. Sundip Charmode |
| Step 2 | Clinical case-based discussion | 10 minutes | Anatomy | Dr. Sundip Charmode |

**Step 2 – Case scenario:** A 26-year-old male presents with a gradually increasing, painless swelling in the right lower abdomen, which becomes more prominent when standing or coughing and reduces when lying down. On examination, a soft, reducible swelling is palpated in the right inguinal region with a positive cough impulse. What is the likely diagnosis? What are the other possible differential diagnoses? Mention the anatomical basis for this condition along with the possible complications.

**Step 3 – Group activity:** Roll numbers 6–10 [Dissection hall]. Structural model making on '***Inguinal canal showing normal anatomy and inguinal hernia***'.

**Post-test:**

- Models will be evaluated using the Model Grading Scale and rated on creativity, communication skills, and problem-solving accuracy. [Dr. Sundip Charmode / other Anatomy faculty]
- Application of metacognitive skills in problem-solving tasks will be assessed using a self-designed Creative Problem-Solving Scale. [Dr. Sundip Charmode]
- Objective assessment (post-test). [Dr. Sundip Charmode]
- Feedback will be collected from students and faculty (Anatomy) using a Module Feedback Questionnaire. [Dr. Sundip Charmode]

**Instructions for the group activity (model-making task):**

- Roles and responsibilities: All group members should assign distinct roles and responsibilities to each other for the task.
- Materials: Use eco-friendly materials, such as clay, cardboard, or colored wires for blood vessels, muscles, bones, cartilages and related structures.
- Anatomical accuracy: Label the structures forming the boundaries, inlet and outlet of the inguinal canal. Label the various coverings of the hernial sac.
- Creativity: Use color codes and creative elements to distinguish boundaries, contents, inlet and outlet of the inguinal canal along with the coverings of the hernial sac.
- Submit by the deadline given; a presentation will be conducted on the date specified.

## Module 3: Pelvic diaphragm

**Aim:** The learner should be able to describe the anatomical position, components, and description of muscles (origin, insertion, action, nerve supply), openings, functions and applied anatomy of the pelvic diaphragm.

**Objectives:**

- To enhance problem-solving skills required to understand the anatomical basis of pathologies affecting the pelvic diaphragm.
- To enhance collaborative and creative skills necessary for effective participation in a group activity like 'model making task'.

**Pre-test:**

- Assessment of metacognitive awareness using the Metacognition Awareness Inventory (MAI), a 52-item scale. [Dr. Sundip Charmode]
- Objective assessment of students' baseline knowledge of the anatomical position, components, description of muscles, nerve supply, openings, functions, and applied anatomy of the pelvic diaphragm. [Dr. Sundip Charmode]

**Module contents:** The module consists of 3 steps.

- Step 1 and Step 2 will be conducted as an integrated session in the lecture theatre.
- Step 3 will be a group activity (model-making task) conducted in the dissection hall.

*Step 1 and 2: Integrated session (55 minutes)*

| Step | Topic / session | Duration | Department | Faculty |
|---|---|---|---|---|
| Step 1 | Gross anatomy of pelvic diaphragm | 20 minutes | Anatomy | Dr. Sundip Charmode |
| | Applied anatomy of pelvic diaphragm | 15 minutes | Obstetrics and Gynecology | – |
| | Application of metacognition skills and strategies | 10 minutes | Anatomy | Dr. Sundip Charmode |
| Step 2 | Clinical case-based discussion | 10 minutes | Anatomy | Dr. Sundip Charmode |

**Step 2 – Case scenario:** A 45-year-old female, who recently gave birth to her third child, presents with complaints of pelvic heaviness, involuntary urine leakage during coughing, and a sensation of something bulging in the vaginal area. Clinical examination reveals a prolapsed pelvic organ and weakened pelvic floor muscles. Identify the condition. Discuss the possible deficiencies in the pelvic diaphragm and their anatomical basis.

**Step 3 – Group activity:** Roll numbers 11–15 [Dissection hall]. Model making on '***Pelvic diaphragm showing uterine and vaginal prolapse***'.

**Post-test:**

- Models will be evaluated using the Model Grading Scale and rated on creativity, communication skills, and problem-solving accuracy. [Dr. Sundip Charmode / other Anatomy faculty]
- Application of metacognitive skills in problem-solving tasks will be assessed using a self-designed Creative Problem-Solving Scale. [Dr. Sundip Charmode]
- Objective assessment (post-test). [Dr. Sundip Charmode]
- Feedback will be collected from students and faculty (Anatomy) using a Module Feedback Questionnaire. [Dr. Sundip Charmode]

**Instructions for the group activity (model-making task):**

- Roles and responsibilities: All group members should assign distinct roles and responsibilities to each other for the task.
- Materials: Use eco-friendly materials, such as clay, cardboard, or colored wires for blood vessels, muscles, bones, cartilages and related structures.
- Anatomical accuracy: Label the various components, muscles, and openings present in the pelvic diaphragm.
- Creativity: Use color codes and creative elements to distinguish muscles, bones, and structures opening into the pelvic diaphragm.
- Submit by the deadline given; a presentation will be conducted on the date specified.

## Module 4: Femoral triangle

**Aim:** The learner should be able to describe the anatomical position, boundaries, contents, and applied anatomy of the femoral triangle.

**Objectives:**

- To enhance problem-solving skills required to understand the anatomical basis of pathologies affecting the femoral triangle.
- To enhance collaborative and creative skills necessary for effective participation in a group activity like 'model making task'.

**Pre-test:**

- Assessment of metacognitive awareness using the Metacognition Awareness Inventory (MAI), a 52-item scale. [Dr. Sundip Charmode]
- Objective assessment of students' baseline knowledge of the boundaries, contents, and applied anatomy of the femoral triangle. [Dr. Sundip Charmode]

**Module contents:** The module consists of 3 steps.

- Step 1 and Step 2 will be conducted as an integrated session in the lecture theatre.
- Step 3 will be a group activity (model-making task) conducted in the dissection hall.

*Step 1 and 2: Integrated session (55 minutes)*

| Step | Topic / session | Duration | Department | Faculty |
|---|---|---|---|---|
| Step 1 | Gross anatomy of femoral triangle | 20 minutes | Anatomy | Dr. Sundip Charmode |
| | Applied anatomy of femoral triangle | 15 minutes | General Surgery | – |
| | Application of metacognition skills and strategies | 10 minutes | Anatomy | Dr. Sundip Charmode |
| Step 2 | Clinical case-based discussion | 10 minutes | Anatomy | Dr. Sundip Charmode |

**Step 2 – Case scenario:** A 60-year-old female presents with a painful swelling in the right groin, which worsens with standing and lifting heavy objects and improves with rest. On clinical examination, a firm, non-reducible bulge is felt below the inguinal ligament, suggesting a hernial sac. What is the most likely diagnosis, and what are the differential diagnoses? Mention its anatomical basis and the possible complications of this condition.

**Step 3 – Group activity:** Roll numbers 16–20 [Dissection hall]. Structural model making on '***Femoral canal: boundaries and contents, showing femoral hernia***'.

**Post-test:**

- Models will be evaluated using the Model Grading Scale and rated on creativity, communication skills, and problem-solving accuracy. [Dr. Sundip Charmode / other Anatomy faculty]
- Application of metacognitive skills in problem-solving tasks will be assessed using a self-designed Creative Problem-Solving Scale. [Dr. Sundip Charmode]
- Objective assessment (post-test). [Dr. Sundip Charmode]
- Feedback will be collected from students and faculty (Anatomy) using a Module Feedback Questionnaire. [Dr. Sundip Charmode]

**Instructions for the group activity (model-making task):**

- Roles and responsibilities: All group members should assign distinct roles and responsibilities to each other for the task.
- Materials: Use eco-friendly materials, such as clay, cardboard, or colored wires for blood vessels, muscles, bones, cartilages and related structures.
- Anatomical accuracy: Label the structures forming the boundaries and contents of the femoral canal. Label the various coverings of the hernial sac and mark the direction of its movement.
- Creativity: Use color codes and creative elements to distinguish boundaries and contents.
- Submit by the deadline given; a presentation will be conducted on the date specified.

## Module 5: Arches of foot

**Aim:** The learner should be able to classify and describe the types and composition of the longitudinal and transverse arches of the foot, with their functions and clinical anatomy.

**Objectives:**

- To enhance problem-solving skills required to understand the anatomical basis of pathologies affecting the longitudinal arches of the foot.
- To enhance collaborative and creative skills necessary for effective participation in a group activity like 'model making task'.

**Pre-test:**

- Assessment of metacognitive awareness using the Metacognition Awareness Inventory (MAI), a 52-item scale. [Dr. Sundip Charmode]
- Objective assessment of students' baseline knowledge of the classification and composition of the various arches of the foot, their functions, and clinical anatomy. [Dr. Sundip Charmode]

**Module contents:** The module consists of 3 steps.

- Step 1 and Step 2 will be conducted as an integrated session in the lecture theatre.
- Step 3 will be a group activity (model-making task) conducted in the dissection hall.

*Step 1 and 2: Integrated session (55 minutes)*

| Step | Topic / session | Duration | Department | Faculty |
|---|---|---|---|---|
| Step 1 | Gross anatomy of arches of foot | 20 minutes | Anatomy | Dr. Sundip Charmode |
| | Applied anatomy of arches of foot | 15 minutes | Orthopedics | – |
| | Application of metacognition skills and strategies | 10 minutes | Anatomy | Dr. Sundip Charmode |
| Step 2 | Clinical case-based discussion | 10 minutes | Anatomy | Dr. Sundip Charmode |

**Step 2 – Case scenario:** A 25-year-old male presents with complaints of foot pain and discomfort, particularly after long periods of standing and walking. On examination, there is flattening of the medial arch, and the entire sole touches the ground when standing, with mild ankle pain. What is the likely diagnosis, and what is the anatomical basis of it? What are the possible complications of this condition?

**Step 3 – Group activity:** Roll numbers 21–25 [Dissection hall]. Working model making on '***Longitudinal arches of foot showing flat foot***'.

**Post-test:**

- Models will be evaluated using the Model Grading Scale and rated on creativity, communication skills, and problem-solving accuracy. [Dr. Sundip Charmode / other Anatomy faculty]
- Application of metacognitive skills in problem-solving tasks will be assessed using a self-designed Creative Problem-Solving Scale. [Dr. Sundip Charmode]
- Objective assessment (post-test). [Dr. Sundip Charmode]
- Feedback will be collected from students and faculty (Anatomy) using a Module Feedback Questionnaire. [Dr. Sundip Charmode]

**Instructions for the group activity (model-making task):**

- Roles and responsibilities: All group members should assign distinct roles and responsibilities to each other for the task.
- Materials: Use eco-friendly materials, such as clay, cardboard, or colored wires for blood vessels, muscles, tendons, bones, joints, cartilages and related structures.
- Anatomical accuracy: Label the osseo-ligamentous structures forming the longitudinal arches of the foot.
- Creativity: Use color codes and creative elements to distinguish bones, joints, muscles, ligaments and tendons.
- Submit by the deadline given; a presentation will be conducted on the date specified.

## Module 6: Knee joint

**Aim:** The learner should be able to describe the type of joint, articulating bones, intra-articular structures (with their attachments), movements occurring at the joint, muscles causing movements, and applied anatomy of the knee joint.

**Objectives:**

- To enhance problem-solving skills required to understand the anatomical basis of pathologies affecting the intra-articular and extra-articular osseo-ligamentous structures of the knee joint.
- To enhance collaborative and creative skills necessary for effective participation in a group activity like 'model making task'.

**Pre-test:**

- Assessment of metacognitive awareness using the Metacognition Awareness Inventory (MAI), a 52-item scale. [Dr. Sundip Charmode]
- Objective assessment of students' baseline knowledge of the type of joint, articulating bones, intra-articular structures, movements at the joint, muscles causing movements, and applied anatomy of the knee joint. [Dr. Sundip Charmode]

**Module contents:** The module consists of 3 steps.

- Step 1 and Step 2 will be conducted as an integrated session in the lecture theatre.
- Step 3 will be a group activity (model-making task) conducted in the dissection hall.

*Step 1 and 2: Integrated session (55 minutes)*

| Step | Topic / session | Duration | Department | Faculty |
|---|---|---|---|---|
| Step 1 | Gross anatomy of knee joint | 20 minutes | Anatomy | Dr. Sundip Charmode |
| | Applied anatomy of knee joint | 15 minutes | Orthopedics | – |
| | Application of metacognition skills and strategies | 10 minutes | Anatomy | Dr. Sundip Charmode |
| Step 2 | Clinical case-based discussion | 10 minutes | Anatomy | Dr. Sundip Charmode |

**Step 2 – Case scenario:** A 30-year-old male presents with sudden knee pain and swelling after turning during a soccer game, accompanied by a popping sound at the time of injury. On examination, there is tenderness around the knee joint, decreased range of motion, and a positive Lachman test. What is the most likely diagnosis, and what are the other differential diagnoses in this patient? What are the possible complications if this condition is left untreated?

**Step 3 – Group activity:** Roll numbers 26–30 [Dissection hall]. Structural model making on '***Knee joint showing all the intra-articular structures***'.

**Post-test:**

- Models will be evaluated using the Model Grading Scale and rated on creativity, communication skills, and problem-solving accuracy. [Dr. Sundip Charmode / other Anatomy faculty]
- Application of metacognitive skills in problem-solving tasks will be assessed using a self-designed Creative Problem-Solving Scale. [Dr. Sundip Charmode]
- Objective assessment (post-test). [Dr. Sundip Charmode]
- Feedback will be collected from students and faculty (Anatomy) using a Module Feedback Questionnaire. [Dr. Sundip Charmode]

**Instructions for the group activity (model-making task):**

- Roles and responsibilities: All group members should assign distinct roles and responsibilities to each other for the task.
- Materials: Use eco-friendly materials, such as clay, cardboard, or colored wires for blood vessels, nerves, ligaments, muscles, bones, cartilages and other related structures.
- Anatomical accuracy: Label the articulating bones and intra-articular structures of the knee joint.
- Creativity: Use color codes and creative elements to distinguish bones, cartilages and ligaments.
- Submit by the deadline given; a presentation will be conducted on the date specified.

## Module 7: Facial nerve

**Aim:** The learner should be able to describe the origin (central and peripheral), functional components, course (intracranial and extracranial), relations, branches and anatomy of the facial nerve.

**Objectives:**

- To enhance problem-solving skills required to understand the various sites of compression of the facial nerve along its course and its different clinical presentations.
- To enhance collaborative and creative skills necessary for effective participation in a group activity like 'model making task'.

**Pre-test:**

- Assessment of metacognitive awareness using the Metacognition Awareness Inventory (MAI), a 52-item scale. [Dr. Sundip Charmode]
- Objective assessment of students' baseline knowledge of the origin, functional components, course, relations, branches, and applied anatomy of the facial nerve. [Dr. Sundip Charmode]

**Module contents:** The module consists of 3 steps.

- Step 1 and Step 2 will be conducted as an integrated session in the lecture theatre.
- Step 3 will be a group activity (model-making task) conducted in the dissection hall.

*Step 1 and 2: Integrated session (55 minutes)*

| Step | Topic / session | Duration | Department | Faculty |
|---|---|---|---|---|
| Step 1 | Gross anatomy of facial nerve | 20 minutes | Anatomy | Dr. Sundip Charmode |
| | Applied anatomy of facial nerve | 15 minutes | Medicine | – |
| | Application of metacognition skills and strategies | 10 minutes | Anatomy | Dr. Sundip Charmode |
| Step 2 | Clinical case-based discussion | 10 minutes | Anatomy | Dr. Sundip Charmode |

**Step 2 – Case scenario:** A 45-year-old female presents with sudden onset of facial weakness, difficulty closing her right eye, and drooping of the right side of her mouth, occurring over the past 24 hours. On examination, there is decreased sensation and weakness of the facial muscles on the right side, with no other neurological deficits. What is the most likely diagnosis? Mention the different sites of compression of the facial nerve along its course, with its varying presentations.

**Step 3 – Group activity:** Roll numbers 31-35 * [Dissection hall]. Structural model making on '***Facial nerve: course and distribution in the intracranial cavity and face***'.

**Post-test:**

- Models will be evaluated using the Model Grading Scale and rated on creativity, communication skills, and problem-solving accuracy. [Dr. Sundip Charmode / other Anatomy faculty]
- Application of metacognitive skills in problem-solving tasks will be assessed using a self-designed Creative Problem-Solving Scale. [Dr. Sundip Charmode]
- Objective assessment (post-test). [Dr. Sundip Charmode]
- Feedback will be collected from students and faculty (Anatomy) using a Module Feedback Questionnaire. [Dr. Sundip Charmode]

**Instructions for the group activity (model-making task):**

- Roles and responsibilities: All group members should assign distinct roles and responsibilities to each other for the task.
- Materials: Use eco-friendly materials, such as clay, cardboard, or colored wires for blood vessels, muscles, bones, cartilages and related structures.
- Anatomical accuracy: Label the various central and peripheral nuclei, branches, bony canals, and other relations of the facial nerve during its course within and outside the cranium.
- Creativity: Use color codes and creative elements to distinguish the various central and peripheral nuclei, branches, and areas of distribution.
- Submit by the deadline given; a presentation will be conducted on the date specified.

**Aim:** The learner should be able to describe the parts of the ventricular system, communications, related structures, CSF formation, circulation and absorption, functions of CSF, and its applied anatomy.

**Objectives:**

- To enhance problem-solving skills required to understand the various sites of obstruction in CSF circulation, its causes, and different clinical presentations.
- To enhance collaborative and creative skills necessary for effective participation in a group activity like 'model making task'.

**Pre-test:**

- Assessment of metacognitive awareness using the Metacognition Awareness Inventory (MAI), a 52-item scale. [Dr. Sundip Charmode]
- Objective assessment of students' baseline knowledge of the anatomy and parts of the ventricular system, CSF formation, circulation and absorption, functions of CSF, and its applied anatomy. [Dr. Sundip Charmode]

**Module contents:** The module consists of 3 steps.

- Step 1 and Step 2 will be conducted as an integrated session in the lecture theatre.
- Step 3 will be a group activity (model-making task) conducted in the dissection hall.

*Step 1 and 2: Integrated session (55 minutes)*

| Step | Topic / session | Duration | Department | Faculty |
|---|---|---|---|---|
| Step 1 | Gross anatomy of CSF circulation | 20 minutes | Anatomy | Dr. Sundip Charmode |
| | Applied anatomy of CSF circulation | 15 minutes | Medicine | – |
| | Application of metacognition skills and strategies | 10 minutes | Anatomy | Dr. Sundip Charmode |
| Step 2 | Clinical case-based discussion | 10 minutes | Anatomy | Dr. Sundip Charmode |

**Step 2 – Case scenario:** A 3-month-old infant presents with a rapidly increasing head circumference, bulging fontanelles, and irritability. On examination, the infant shows signs of delayed developmental milestones and a positive "sunsetting sign". What is the likely diagnosis and its different types? What are the main steps in diagnosing this condition and its differential diagnosis? What complications are likely to occur if this condition is left untreated?

**Step 3 – Group activity:** Roll numbers 36-40 * [Dissection hall]. Model making on '***Ventricular system showing the lateral, third and fourth ventricles along with their communications and CSF circulation***'.

**Post-test:**

- Models will be evaluated using the Model Grading Scale and rated on creativity, communication skills, and problem-solving accuracy. [Dr. Sundip Charmode / other Anatomy faculty]
- Application of metacognitive skills in problem-solving tasks will be assessed using a self-designed Creative Problem-Solving Scale. [Dr. Sundip Charmode]
- Objective assessment (post-test). [Dr. Sundip Charmode]
- Feedback will be collected from students and faculty (Anatomy) using a Module Feedback Questionnaire. [Dr. Sundip Charmode]

**Instructions for the group activity (model-making task):**

- Roles and responsibilities: All group members should assign distinct roles and responsibilities to each other for the task.
- Materials: Use eco-friendly materials, such as clay, cardboard, or colored wires for blood vessels, muscles, bones, cartilages and related structures.
- Anatomical accuracy: Label the various parts of the ventricular system and its communications, along with their boundaries and related structures. Also show the direction of CSF circulation.
- Creativity: Use color codes and creative elements to distinguish the different parts of the ventricular system.
- Submit by the deadline given; a presentation will be conducted on the date specified.

**Aim:** The learner should be able to describe the anatomical position, skeleton of the larynx (cartilages and muscles), laryngeal cavity and its subdivisions, movements of the rima glottidis, blood supply, nerve supply, lymphatic drainage, and applied anatomy of the larynx.

**Objectives:**

- To enhance problem-solving skills required to understand the anatomical basis of pathologies affecting the cartilages, muscles, and structures of the laryngeal cavity, along with related neurovascular structures and their varying presentations.
- To enhance collaborative and creative skills necessary for effective participation in a group activity like 'model making task'.

**Pre-test:**

- Assessment of metacognitive awareness using the Metacognition Awareness Inventory (MAI), a 52-item scale. [Dr. Sundip Charmode]
- Objective assessment of students' baseline knowledge of the skeleton of the larynx, laryngeal cavity and its subdivisions, movements of the rima glottidis, blood supply, nerve supply, lymphatic drainage, and applied anatomy of the larynx. [Dr. Sundip Charmode]

**Module contents:** The module consists of 3 steps.

- Step 1 and Step 2 will be conducted as an integrated session in the lecture theatre.
- Step 3 will be a group activity (model-making task) conducted in the dissection hall.

*Step 1 and 2: Integrated session (55 minutes)*

| Step | Topic / session | Duration | Department | Faculty |
|---|---|---|---|---|
| Step 1 | Gross anatomy of larynx | 20 minutes | Anatomy | Dr. Sundip Charmode |
| | Applied anatomy of larynx | 15 minutes | ENT | – |
| | Application of metacognition skills and strategies | 10 minutes | Anatomy | Dr. Sundip Charmode |
| Step 2 | Clinical case-based discussion | 10 minutes | Anatomy | Dr. Sundip Charmode |

**Step 2 – Case scenario:** A 45-year-old male presents with hoarseness of voice, difficulty in swallowing, and occasional choking episodes over the past 3 weeks. On examination, there is reduced movement of the right vocal cord during phonation, with normal sensation in the larynx. What is the most likely diagnosis, and what are the potential causes? Mention the differential diagnosis for this condition.

**Step 3 – Group activity:** Roll numbers 41–45 * [Dissection hall]. Structural model making on '***Larynx, showing the articulation of cartilages and muscles, the laryngeal cavity and its subdivisions, and movements of the rima glottidis***'.

**Post-test:**

- Models will be evaluated using the Model Grading Scale and rated on creativity, communication skills, and problem-solving accuracy. [Dr. Sundip Charmode / other Anatomy faculty]
- Application of metacognitive skills in problem-solving tasks will be assessed using a self-designed Creative Problem-Solving Scale. [Dr. Sundip Charmode]
- Objective assessment (post-test). [Dr. Sundip Charmode]
- Feedback will be collected from students and faculty (Anatomy) using a Module Feedback Questionnaire. [Dr. Sundip Charmode]

**Instructions for the group activity (model-making task):**

- Roles and responsibilities: All group members should assign distinct roles and responsibilities to each other for the task.
- Materials: Use eco-friendly materials, such as clay, cardboard, or colored wires for ligaments, mucosal folds, blood vessels, muscles, bones, cartilages and related structures.
- Anatomical accuracy: Label the various cartilages, muscles, ligaments, and folds of the laryngeal cavity, along with related neurovascular structures.
- Creativity: Use color codes and creative elements to distinguish cartilages, muscles, ligaments, mucosal folds, structures in the laryngeal cavity, and neurovascular structures.
- Submit by the deadline given; a presentation will be conducted on the date specified.

## Module 10: Tympanic cavity (Middle ear)

**Aim:** The learner should be able to describe the anatomical position, subdivisions, boundaries, contents, communications, blood supply, nerve supply, and applied anatomy of the tympanic cavity.

**Objectives:**

- To enhance problem-solving skills required to understand the anatomical basis of pathologies affecting the boundaries and contents of the tympanic cavity, with their possible complications and clinical presentations.
- To enhance collaborative and creative skills necessary for effective participation in a group activity like 'model making task'.

**Pre-test:**

- Assessment of metacognitive awareness using the Metacognition Awareness Inventory (MAI), a 52-item scale. [Dr. Sundip Charmode]
- Objective assessment of students' baseline knowledge of the subdivisions, boundaries, contents, communications, blood supply, nerve supply, and applied anatomy of the tympanic cavity. [Dr. Sundip Charmode]

**Module contents:** The module consists of 3 steps.

- Step 1 and Step 2 will be conducted as an integrated session in the lecture theatre.
- Step 3 will be a group activity (model-making task) conducted in the dissection hall.

*Step 1 and 2: Integrated session (55 minutes)*

| Step | Topic / session | Duration | Department | Faculty |
|---|---|---|---|---|
| Step 1 | Gross anatomy of tympanic cavity | 20 minutes | Anatomy | Dr. Sundip Charmode |
| | Applied anatomy of tympanic cavity | 15 minutes | ENT | – |
| | Application of metacognition skills and strategies | 10 minutes | Anatomy | Dr. Sundip Charmode |
| Step 2 | Clinical case-based discussion | 10 minutes | Anatomy | Dr. Sundip Charmode |

**Step 2 – Case scenario:** A 6-year-old child presents with ear pain, fever, and irritability for the past 2 days, along with difficulty hearing in the affected ear. On examination, the tympanic membrane is red, bulging, and immobile, with tenderness over the mastoid process. What is the most likely diagnosis and other differential diagnoses? What can be the possible complications of this condition?

**Step 3 – Group activity:** Roll numbers 46–50 [Dissection hall]. Structural model making on '***Tympanic cavity showing its boundaries, relations, contents and communications***'.

**Post-test:**

- Models will be evaluated using the Model Grading Scale and rated on creativity, communication skills, and problem-solving accuracy. [Dr. Sundip Charmode / other Anatomy faculty]
- Application of metacognitive skills in problem-solving tasks will be assessed using a self-designed Creative Problem-Solving Scale. [Dr. Sundip Charmode]
- Objective assessment (post-test). [Dr. Sundip Charmode]
- Feedback will be collected from students and faculty (Anatomy) using a Module Feedback Questionnaire. [Dr. Sundip Charmode]

**Instructions for the group activity (model-making task):**

- Roles and responsibilities: All group members should assign distinct roles and responsibilities to each other for the task.
- Materials: Use eco-friendly materials, such as clay, cardboard, or colored wires for blood vessels, muscles, bones, cartilages and related structures.
- Anatomical accuracy: Label the various subdivisions, boundaries, contents, and communications of the tympanic cavity, along with the various arteries, veins and nerves entering and exiting it.
- Creativity: Use color codes and creative elements to distinguish boundaries and contents, and the related neurovascular structures.
- Submit by the deadline given; a presentation will be conducted on the date specified.

## References


1. Schraw G, Dennison RS. Assessing metacognitive awareness. Contemp Educ Psychol. 1994;19(4):460–475. https://doi.org/10.1006/ceps.1994.1033.
2. Urban K, Urban M. How can we measure metacognition in creative problem-solving? Standardization of the MCPS scale. Think Skills Creat. 2023;49:101345.
3. Williams R, Brennan J. Collecting and using student feedback: a guide to good practice. York, UK: Higher Education Academy; 2004.

# Supplementary file 6

## Compilation of Questionnaires and Rating Scales Used in the CPSSM Study

**Author:** Dr. Sundip Charmode, Dr. Simmi Mehra, Prof. Ciraj Ali Mohammed, Prof. Abdul Ajees Abdul Salam

*This file compiles, in full, all six questionnaires and rating scales used during the Creative Problem-Solving Skills Module (CPSSM): one published metacognition inventory used as a pre-test, two published creativity/metacognition scales, one self-designed group-process scale, one self-designed model scoring rubric used for post-test assessment, and one self-designed module feedback questionnaire. Published instruments are reproduced for research/educational documentation purposes with full citation; self-designed instruments were developed by the author for this study. References cited within this file are listed in a single, consolidated reference list at the end and are not repeated in the main manuscript reference list.*

---

## Instrument 1. Metacognitive Awareness Inventory (MAI) — used as pre-test

Think of yourself as a learner. Read each statement carefully. Consider whether the statement is true or false as it generally applies to you when you are in the role of a learner (student, attending classes, university, etc.). Mark True or False as appropriate for each of the 52 items below.

| No. | Item | True | False |
|---|---|---|---|
| 1 | I ask myself periodically if I am meeting my goals. | | |
| 2 | I consider several alternatives to a problem before I answer. | | |
| 3 | I try to use strategies that have worked in the past. | | |
| 4 | I pace myself while learning in order to have enough time. | | |
| 5 | I understand my intellectual strengths and weaknesses. | | |
| 6 | I think about what I really need to learn before I begin a task. | | |
| 7 | I know how well I did once I finish a test. | | |
| 8 | I set specific goals before I begin a task. | | |
| 9 | I slow down when I encounter important information. | | |
| 10 | I know what kind of information is most important to learn. | | |
| 11 | I ask myself if I have considered all options when solving a problem. | | |
| 12 | I am good at organizing information. | | |
| 13 | I consciously focus my attention on important information. | | |
| 14 | I have a specific purpose for each strategy I use. | | |
| 15 | I learn best when I know something about the topic. | | |
| 16 | I know what the teacher expects me to learn. | | |
| 17 | I am good at remembering information. | | |
| 18 | I use different learning strategies depending on the situation. | | |
| 19 | I ask myself if there was an easier way to do things after I finish a task. | | |
| 20 | I have control over how well I learn. | | |
| 21 | I periodically review to help me understand important relationships. | | |
| 22 | I ask myself questions about the material before I begin. | | |
| 23 | I think of several ways to solve a problem and choose the best one. | | |

| | | | |
|---|---|---|---|
| 24 | I summarize what I've learned after I finish. | | |
| 25 | I ask others for help when I don't understand something. | | |
| 26 | I can motivate myself to learn when I need to. | | |
| 27 | I am aware of what strategies I use when I study. | | |
| 28 | I find myself analyzing the usefulness of strategies while I study. | | |
| 29 | I use my intellectual strengths to compensate for my weaknesses. | | |
| 30 | I focus on the meaning and significance of new information. | | |
| 31 | I create my own examples to make information more meaningful. | | |
| 32 | I am a good judge of how well I understand something. | | |
| 33 | I find myself using helpful learning strategies automatically. | | |
| 34 | I find myself pausing regularly to check my comprehension. | | |
| 35 | I know when each strategy I use will be most effective. | | |
| 36 | I ask myself how well I accomplish my goals once I'm finished. | | |
| 37 | I draw pictures or diagrams to help me understand while learning. | | |
| 38 | I ask myself if I have considered all options after I solve a problem. | | |
| 39 | I try to translate new information into my own words. | | |
| 40 | I change strategies when I fail to understand. | | |
| 41 | I use the organizational structure of the text to help me learn. | | |
| 42 | I read instructions carefully before I begin a task. | | |
| 43 | I ask myself if what I'm reading is related to what I already know. | | |
| 44 | I reevaluate my assumptions when I get confused. | | |
| 45 | I organize my time to best accomplish my goals. | | |
| 46 | I learn more when I am interested in the topic. | | |
| 47 | I try to break studying down into smaller steps. | | |
| 48 | I focus on overall meaning rather than specifics. | | |
| 49 | I ask myself questions about how well I am doing while I am learning something new. | | |
| 50 | I ask myself if I learned as much as I could have once I finish a task. | | |
| 51 | I stop and go back over new information that is not clear. | | |
| 52 | I stop and reread when I get confused. | | |

## Scoring guide

For each "True" response, score 1 point; for each "False" response, score 0 points. Sum the points within each subscale below.

## Knowledge of cognition

- Declarative knowledge: factual knowledge the learner needs before being able to process or use critical thinking related to the topic; knowledge of one's skills, intellectual resources, and abilities as a learner.
- Procedural knowledge: the application of knowledge for the purpose of completing a procedure or process; knowledge of how to implement learning strategies.
- Conditional knowledge: knowledge of when and why to use particular learning procedures; application of declarative and procedural knowledge under specific conditions.

| No. | Declarative knowledge items |
|---|---|
| 5 | I understand my intellectual strengths and weaknesses. |
| 10 | I know what kind of information is most important to learn. |
| 12 | I am good at organizing information. |
| 16 | I know what the teacher expects me to learn. |
| 17 | I am good at remembering information. |
| 20 | I have control over how well I learn. |
| 32 | I am a good judge of how well I understand something. |
| 46 | I learn more when I am interested in the topic. |
| | **Maximum subscale score: 8** |
| **No.** | **Procedural knowledge items** |
| 3 | I try to use strategies that have worked in the past. |
| 14 | I have a specific purpose for each strategy I use. |
| 27 | I am aware of what strategies I use when I study. |
| 33 | I find myself using helpful learning strategies automatically. |
| | **Maximum subscale score: 4** |
| **No.** | **Conditional knowledge items** |
| 15 | I learn best when I know something about the topic. |
| 18 | I use different learning strategies depending on the situation. |
| 26 | I can motivate myself to learn when I need to. |
| 29 | I use my intellectual strengths to compensate for my weaknesses. |
| 35 | I know when each strategy I use will be most effective. |
| | **Maximum subscale score: 5** |

### Regulation of cognition

- Planning: goal setting and allocating resources prior to learning.
- Information management strategies: skills and strategy sequences used to process information more efficiently (organizing, elaborating, summarizing, selective focusing).
- Comprehension monitoring: assessment of one's own learning or strategy use.
- Debugging strategies: strategies used to correct comprehension and performance errors.
- Evaluation: analysis of performance and strategy effectiveness after a learning episode.

| No. | Planning items |
|---|---|
| 4 | I pace myself while learning in order to have enough time. |
| 6 | I think about what I really need to learn before I begin a task. |
| 8 | I set specific goals before I begin a task. |
| 22 | I ask myself questions about the material before I begin. |
| 23 | I think of several ways to solve a problem and choose the best one. |
| 42 | I read instructions carefully before I begin a task. |
| 45 | I organize my time to best accomplish my goals. |
| | **Maximum subscale score: 7** |
| **No.** | **Information management items** |
| 9 | I slow down when I encounter important information. |
| 13 | I consciously focus my attention on important information. |

| | |
|---|---|
| 30 | I focus on the meaning and significance of new information. |
| 31 | I create my own examples to make information more meaningful. |
| 37 | I draw pictures or diagrams to help me understand while learning. |
| 39 | I try to translate new information into my own words. |
| 41 | I use the organizational structure of the text to help me learn. |
| 43 | I ask myself if what I'm reading is related to what I already know. |
| 47 | I try to break studying down into smaller steps. |
| 48 | I focus on overall meaning rather than specifics. |
| | **Maximum subscale score: 10** |
| **No.** | **Comprehension monitoring items** |
| 1 | I ask myself periodically if I am meeting my goals. |
| 2 | I consider several alternatives to a problem before I answer. |
| 11 | I ask myself if I have considered all options when solving a problem. |
| 21 | I periodically review to help me understand important relationships. |
| 28 | I find myself analyzing the usefulness of strategies while I study. |
| 34 | I find myself pausing regularly to check my comprehension. |
| 49 | I ask myself questions about how well I am doing while I am learning something new. |
| | **Maximum subscale score: 7** |
| **No.** | **Debugging strategies items** |
| 25 | I ask others for help when I don't understand something. |
| 40 | I change strategies when I fail to understand. |
| 44 | I reevaluate my assumptions when I get confused. |
| 51 | I stop and go back over new information that is not clear. |
| 52 | I stop and reread when I get confused. |
| | **Maximum subscale score: 5** |
| **No.** | **Evaluation items** |
| 7 | I know how well I did once I finish a test. |
| 19 | I ask myself if there was an easier way to do things after I finish a task. |
| 24 | I summarize what I've learned after I finish. |
| 36 | I ask myself how well I accomplish my goals once I'm finished. |
| 38 | I ask myself if I have considered all options after I solve a problem. |
| 50 | I ask myself if I learned as much as I could have once I finish a task. |
| | **Maximum subscale score: 6** |

*Source: Schraw G, Dennison RS. Assessing metacognitive awareness. Contemp Educ Psychol. 1994;19(4):460–475. Reproduced for research documentation purposes; see consolidated reference list.*

## Instrument 2. Metacognition in Creative Problem-Solving Scale (MCPS, 11 items)

| No. | Item | Process |
|---|---|---|
| 1 | When I'm working on a project, I ask myself whether what I am doing is leading to the fulfilment of my goal. | MON |
| 2 | When I cannot come up with an original approach to a problem, I go back to the assignment and try to figure it out. | MON/REG* |
| 3 | When I encounter difficulties while solving the problem, I change the way I proceed. | MON/REG |
| 4 | Before I start solving a project assignment, I consider how to approach the problem in an original way. | PLAN* |
| 5 | I often ask myself questions to make sure I am approaching my goal. | MON |
| 6 | When solving an assignment, I try to adapt my approach to best fulfil the requirements. | REG |
| 7 | When solving a problem, I try to think innovatively about the topic instead of just doing the same thing over and over again. | REG* |
| 8 | When preparing for an assignment, I try to identify potential problems or things I don't understand well. | PLAN |
| 9 | When I finish working on an essay or a project, I ask myself if my solution has met my goal. | EVAL |
| 10 | Before I start writing an essay or solving a project assignment, I always set up the steps to achieve my goal. | PLAN |
| 11 | When I finish working on an essay or project, I ask myself if my solution is more original than the solutions of others. | EVAL* |

*PLAN = planning; MON = metacognitive monitoring; REG = metacognitive regulation; EVAL = evaluation; * denotes items that focus on novelty. Each item is answered on a 5-point Likert scale (5 = Strongly agree, 4 = Agree, 3 = Neutral, 2 = Disagree, 1 = Strongly disagree).*

*Source: Urban K, Urban M. How can we measure metacognition in creative problem-solving? Standardization of the MCPS scale. Think Skills Creat. 2023;49:101345.*

## Instrument 3. Short Scale of Self-Creativity (SSCS, 11 items)

Composed of 11 items measuring Creative Self-Efficacy (CSE) and Creative Personal Identity (CPI).

| No. | Item |
|---|---|
| 1 | I think I am a creative person. |
| 2 | My creativity is important for who I am. |
| 3 | I know I can efficiently solve even complicated problems. (CSE) |
| 4 | I trust my creative abilities. (CSE) |
| 5 | My imagination and ingenuity distinguish me from my friends. (CSE) |
| 6 | Many times, I have proved that I can cope with difficult situations. (CSE) |
| 7 | Being a creative person is important to me. |
| 8 | I am sure I can deal with problems requiring creative thinking. (CSE) |
| 9 | I am good at proposing original solutions to problems. (CSE) |
| 10 | Creativity is an important part of myself. |
| 11 | Ingenuity is a characteristic which is important to me. |

*Each item is answered on a 7-point Likert scale (1 = definitely not, 7 = definitely yes).*

*Source: Karwowski M, Lebuda I. Measuring creative self-efficacy and creative personal identity. Int J Creat Probl Solving. 2018;28(1):45–57.*

## Instrument 4. Group Metacognition Scale (GMS) and Teamwork Satisfaction Scale

### Group metacognition scale — Knowledge of cognition

- K1. We know our strengths as learners.
- K2. We know how to select relevant information.
- K3. We know how to use the material.
- K4. We know how to organize the new information.
- K5. We know how to connect new information with prior knowledge.

### Group metacognition scale — Planning

- P1. We plan the learning activities.
- P2. We determine what the task requires.
- P3. We select the appropriate discussion tools.
- P4. We identify the discussion strategies depending on the task.
- P5. We organize our time depending on the task.

### Group metacognition scale — Evaluating

- E1. We make judgments on the workload.
- E2. We make judgments on the instruments.
- E3. We make judgments on our learning outcomes.
- E4. We make judgments on the teamwork process.

### Group metacognition scale — Monitoring

- M1. We ask questions to check our understanding.
- M2. We check our approach to improve our outcomes.
- M3. We improve our work with group processes.
- M4. We detect and correct errors during group processes.

### Teamwork satisfaction scale

- S1. I like to work in a collaborative group with my teammates.
- S2. I like to solve problems with my teammates in group discussions.
- S3. Interacting with the other team members can increase my motivation to learn.
- S4. I have benefited from interacting with my teammates.
- S5. I have benefited from my teammates' feedback.
- S6. Online teamwork promotes creativity.
- S7. Working with my team helps me produce better discussion quality than working individually.
- S8. My team members are sharing knowledge during the teamwork processes.
- S9. I gain online collaboration skills from the teamwork processes.

*All items are answered on a 5-point Likert scale (5 = Strongly agree, 4 = Agree, 3 = Neutral, 2 = Disagree, 1 = Strongly disagree). GMS = Group Metacognition Scale.*

*Source: Chao CT, Chiu YL, Tsai CL, et al. Identifying group metacognition associated with medical students' teamwork satisfaction in an online small group tutorial context. BMC Med Educ. 2024;24:1114. https://doi.org/10.1186/s12909-024-06116-4. Reproduced/adapted for research documentation purposes; see consolidated reference list.*

## Instrument 5. Model-Making Grading Rubric (self-designed) — used as post-test

Creative problem-solving working models were assessed by faculty raters against the following 11 criteria, scored as indicated per criterion (maximum total = 115 marks per model). In this cohort, the ten teams built

models on: (1) Liver, (2) Inguinal canal, (3) Pelvic diaphragm, (4) Femoral triangle, (5) Arches of foot, (6) Knee joint, (7) Facial nerve, (8) Ventricular system and CSF circulation, (9) Larynx, and (10) Tympanic cavity.

- **Anatomical Accuracy (15 marks).** The model accurately represents the anatomical structures, including bones, ligaments, muscles, and their relations.
- **Functionality and Mechanics (15 marks).** The model demonstrates the correct functional anatomy and mimics real-life biomechanics (e.g., joint movements, pressure simulation, deformities).
- **Creativity and Innovation (15 marks).** The model is creatively designed, using innovative materials and techniques to enhance understanding (e.g., movable parts, colour coding, working parts).
- **Clinical Relevance (10 marks).** The model clearly demonstrates clinical conditions or deformities (e.g., disease progression, surgical zones, deformities) and shows practical implications.
- **Teamwork and Collaboration (10 marks).** The team demonstrates clear roles and responsibilities, and effective communication in constructing and presenting the model.
- **Problem-Solving Application (10 marks).** The model addresses a specific challenge or issue (e.g., deformity, muscle weakness, joint movement) and provides solutions or simulations of corrections (e.g., orthotics).
- **Metacognitive Reflection (10 marks).** Each team member provides a thoughtful reflection on their thinking process during the design and construction of the model, including strategies, challenges, and learning outcomes.
- **Presentation Clarity and Explanation (10 marks).** The presentation is clear, concise, and well-organized. The team effectively explains the model's anatomy, function, and relevance, and answers questions.
- **Model Durability and Finish (5 marks).** The model is structurally sound, well-finished, and durable. It should be able to withstand repeated use and clearly demonstrate the concept.
- **Use of Materials and Resourcefulness (5 marks).** The team uses appropriate, available materials to construct a durable and functional model. Creativity in the choice of materials will be considered.
- **Overall Impact and Learning Value (10 marks).** The model provides a comprehensive learning experience for the team and their peers. It should be engaging, educational, and informative.

**Total: 115 marks.**

## Instrument 6. Module Feedback Questionnaire (self-designed)

Please give your feedback regarding your experience of the model-making task on a scale of 1 to 5 (5 = Strongly agree, 4 = Agree, 3 = Neutral, 2 = Disagree, 1 = Strongly disagree).

### A. Questions regarding the overall module

| Question | 5 | 4 | 3 | 2 | 1 | Comments |
|---|---|---|---|---|---|---|
| The module was well organized and met my expectations. | | | | | | |
| The module was clearly structured. | | | | | | |
| Overall, the module was interesting and an enjoyable way of learning. | | | | | | |
| Assessment methods were appropriate and fair. | | | | | | |
| The module has enhanced my understanding of the subject. | | | | | | |
| The module has developed metacognitive, collaborative, and problem-solving skills. | | | | | | |
| The module was relevant to my studies and the teaching methods involved were appropriate for learning. | | | | | | |
| The module was supported by adequate library resources. | | | | | | |
| I have participated actively in seminar/tutorial discussions. | | | | | | |

| | | | | | | |
|---|---|---|---|---|---|---|
| I have spent sufficient time reading/researching to fulfil the module's problem-solving requirements. | | | | | | |
| I would recommend conducting such modules on different subjects. | | | | | | |

### B. Questions regarding the learning experience in Phase 1

| Question | 5 | 4 | 3 | 2 | 1 | Comments |
|---|---|---|---|---|---|---|
| Integrated lectures have been helpful. | | | | | | |
| I have attended all the lectures regularly. | | | | | | |

### C. Questions regarding the importance of the creative problem-solving models (Phase 3)

| Question | 5 | 4 | 3 | 2 | 1 | Comments |
|---|---|---|---|---|---|---|
| The models have greatly enhanced my learning about this topic. | | | | | | |
| The models enhanced my creative skills. | | | | | | |
| The models developed my problem-solving skills. | | | | | | |
| The models developed my ability to work as a team member. | | | | | | |
| Having participated in the problem-solving model making, I feel confident about tackling unfamiliar problems. | | | | | | |
| I am satisfied with all the metacognition measuring scales used in the module. | | | | | | |

### D. Questions regarding the model-making process (Phase 3)

| Question | 5 | 4 | 3 | 2 | 1 | Comments |
|---|---|---|---|---|---|---|
| The understanding and correlation of clinico-radiological and arthro-kinematic findings before model making was difficult. | | | | | | |
| The application of the well-understood findings in creating the problem-solving model was difficult. | | | | | | |
| I had enough time to correlate and understand the patient findings and their application during the model-making process. | | | | | | |
| The model-making process was too heavy. | | | | | | |
| The model-making process was overly theoretical and abstract. | | | | | | |
| There was a lot of pressure on me to do well. | | | | | | |
| The sheer amount of work to go through meant that I could not understand it all thoroughly. | | | | | | |

### E. Questions regarding the collaborative session with the multidisciplinary faculty team (Phase 2)

| Question | 5 | 4 | 3 | 2 | 1 | Comments |
|---|---|---|---|---|---|---|
| The multidisciplinary team members had well-defined objectives regarding the knowledge and skills they expect students to acquire. | | | | | | |
| The multidisciplinary team members guided and motivated me to understand and correlate the project-relevant findings of the patients. | | | | | | |

| | | | | | | |
|---|---|---|---|---|---|---|
| The multidisciplinary team members provided adequate time in instructing and mentoring me in the model-making process. | | | | | | |
| The interactions with the team members were largely constructive and clarified complex concepts in the subject. | | | | | | |

### F. Questions regarding the assessment pattern of the module

| Question | 5 | 4 | 3 | 2 | 1 | Comments |
|---|---|---|---|---|---|---|
| The multidisciplinary team members seemed more interested in testing what I had memorized than what I had understood. | | | | | | |
| The assessment done by the multidisciplinary team was focused more on the functional working of the model. | | | | | | |
| The questionnaire on metacognition skills was meticulously designed and could accurately evaluate my thinking processes. | | | | | | |
| The questionnaire on creativity and self-regulation was precisely directed to evaluate my creative skills. | | | | | | |
| To excel in model making, proficiency in artistic and craft skills is essential. | | | | | | |

### Open-ended questions

- What factors helped you to complete the task of model making?
- What problems did you face while performing the task of model making?
- What factors helped you to collaborate well with the multidisciplinary team and get the most out of the experience?
- What challenges did you encounter while interacting with the multidisciplinary team that limited your learning?
- Comment on up to three aspects of this module which you liked.
- Comment on up to three aspects of this module which you disliked or which could be improved.
- What have you learned in this whole process (module)?
- Would you like to participate in such a method of learning in future?
- Was the time provided for joint, complex model preparation sufficient? Yes/No. If no, please comment.
- Did this module create any hindrance to your routine study? Yes/No. If yes, please comment.
- Was the composition of the multidisciplinary team appropriate? Yes/No. If no, please comment.
- Was the size of the student groups appropriate? Yes/No. If no, please comment.
- Did model making help you in learning anatomy? Yes/No. If no, please comment.
- Can model making be used to replace lectures/practicals for teaching and learning anatomy? Yes/No. If no, please comment.
- Which phase of this module benefited you the most, and what specific skills did it help you improve?
- Any other comments.

## References


1. Chao CT, Chiu YL, Tsai CL, et al. Identifying group metacognition associated with medical students' teamwork satisfaction in an online small group tutorial context. BMC Med Educ. 2024;24:1114. https://doi.org/10.1186/s12909-024-06116-4.
2. Karwowski M, Lebuda I. Measuring creative self-efficacy and creative personal identity. Int J Creat Probl Solving. 2018;28(1):45–57.
3. Schraw G, Dennison RS. Assessing metacognitive awareness. Contemp Educ Psychol. 1994;19(4):460–475. https://doi.org/10.1006/ceps.1994.1033.
4. Urban K, Urban M. How can we measure metacognition in creative problem-solving? Standardization of the MCPS scale. Think Skills Creat. 2023;49:101345.

# SUPPLEMENTARY FILE — Validity and Reliability Data

***Instruments Used (SSCS, MCPS, GMS, Adapted MAI, Model Scoring Scale) and the Module Feedback Questionnaire***

n = 49 (1 of 50 enrolled students excluded — absent at data collection) | Current Study Sample

This supplementary file presents the validity and reliability evidence available for the instruments used in the current study: (1) the Short Scale of Self-Creativity (SSCS); (2) the Metacognition in Creative Problem-Solving Scale (MCPS); (3) the Group Metacognition Scale (GMS), adapted from Chao, Chiu, Tsai, et al. (2024); (4) the adapted Metacognitive Awareness Inventory (MAI); (5) the Model Scoring Scale, a self-designed faculty-rated rubric used as the post-test problem-solving outcome measure; and (6) an investigator-developed Module Feedback Questionnaire. Instruments 1–4 are previously published or previously validated measures (see References); the Model Scoring Scale and Module Feedback Questionnaire were developed for this study, and the Module Feedback Questionnaire's item-level internal consistency was assessed directly in the current sample. Published instruments are reproduced/summarised here for research documentation purposes under the author's stated copyright permission to reuse (not modify) the original items; item wording has not been altered from the source instruments.

*Important note on scope: Content validity (expert-panel I-CVI) and item-level reliability statistics (item-total correlations, factor loadings) for SSCS, MCPS, GMS, and the adapted MAI were established previously — in the original published sources for SSCS and MCPS, and in the companion Phase 2 validation study (Charmode et al., under review) for the adapted GMS and adapted MAI. The dataset for the current study retains only subscale- and total-level summary scores for these instruments rather than each participant's response to every individual item, so item-level statistics could not be recalculated here without re-introducing data that does not exist in the current dataset. Doing so would risk misrepresenting the study's actual evidence base, so this file reports (a) accurate descriptive statistics for these instruments in the current sample, and (b) genuine item-level reliability analysis for the Module Feedback Questionnaire, for which full item-by-item response data were available.*

## Part A: Instrument Descriptive Statistics (Current Sample)

Table 1 reports descriptive statistics for the five instruments listed below (excluding the Module Feedback Questionnaire, covered separately in Part B) as administered/scored in the current study sample (N = 49). Content validity and item-level reliability for the published measures are documented in their original validation sources (SSCS, MCPS) or in the companion Phase 2 study (GMS, adapted MAI; Charmode et al., under review); the Model Scoring Scale is a self-designed rubric with no external validation source. See References.

### Table 1 Descriptive Statistics — Previously Validated Instruments (N = 49)

| Instrument | Construct Measured | Items (k) | Score Range | M | SD | Min–Max | Category Distribution n (%) |
|---|---|---|---|---|---|---|---|
| SSCS | Creative self-efficacy & creative personal identity | 11 | 11–77 | 55.76 | 9.16 | 36–76 | High 6 (12.2); Moderate 38 (77.6); Low 5 (10.2) |
| MCPS (Total) | Metacognition in creative problem-solving | 11 | 11–55 | 44.78 | 3.53 | 34–53 | *Not categorised in current dataset* |
| Adapted MAI (Total) | Metacognitive awareness (knowledge + regulation) | 52 | 0–52 | 38.20 | 5.77 | 25–50 | High 23 (46.9); Moderate 25 (51.0); Low 1 (2.0) |
| GMS (Total) | Group metacognition | 27 | 27–135 | 111.00 | 11.06 | 69–127 | *Not categorised in current dataset* |
| Model Scoring Scale (Post-test) | Faculty-rated problem-solving model quality | 10 criteria | 0–50 | 29.13 | 10.45 | 5–45 | *Not categorised in current dataset* |

*Note. SSCS* = Short Scale of Self-Creativity (Karwowski & Lebuda, 2018); MCPS = Metacognition in Creative Problem-Solving Scale (Urban & Urban, 2023); MAI = Metacognitive Awareness Inventory, scored true/false per item (0–52) following Schraw & Dennison (1994); GMS = Group Metacognition Scale (adapted from Chao, Chiu, Tsai, et al., 2024), rated on a 5-point scale per item (27–135); Model Scoring Scale = self-designed faculty rubric (10 criteria, 5 marks each, total 50) used to score the post-test problem-solving models (see Supplementary File 6 — Compiled Questionnaires, Instrument 5). Category bands follow the cut-points used in the study master data file. GMS, MCPS, and the Model Scoring Scale were not categorised into bands in the current dataset.

### Table 2 Adapted MAI — Sub-Dimension Descriptive Statistics and Composite-Level Internal Consistency (N = 49)

Because only the eight MAI sub-dimension totals (not the 52 individual items) were retained in the current dataset, item-level Cronbach's alpha could not be recalculated. As an exploratory check, internal consistency was instead computed treating the eight sub-dimension scores as components of the total scale.

| MAI Sub-dimension | Items (k) | M | SD | Min | Max |
|---|---|---|---|---|---|
| Declarative knowledge | 8 | 5.59 | 1.21 | 3 | 8 |
| Procedural knowledge | 4 | 2.96 | 0.98 | 1 | 4 |
| Conditional knowledge | 5 | 3.78 | 0.85 | 2 | 5 |
| Planning | 7 | 4.65 | 1.61 | 2 | 7 |
| Information management strategies | 10 | 7.96 | 1.29 | 5 | 10 |
| Comprehension monitoring | 7 | 4.59 | 1.78 | 1 | 7 |
| Debugging strategies | 5 | 4.47 | 0.89 | 1 | 5 |
| Evaluation | 6 | 4.20 | 1.43 | 1 | 6 |

*Note. Composite-level Cronbach's α (8 sub-dimension scores as components) = 0.682, N = 49. This composite-level estimate is conceptually distinct from — and not a substitute for — the full 52-item Cronbach's α (= 0.95) reported for the adapted MAI in the companion Phase 2 validation study (Charmode et al., under review), which was computed from individual item responses. It is reported here only as a supplementary internal-consistency check at the sub-dimension level and should not be cited as the scale's item-level reliability.*

## Part B: Module Feedback Questionnaire — Item-Level Reliability (Current Study, N = 49)

The Module Feedback Questionnaire is an investigator-developed instrument comprising 29 Likert-type items (5-point scale) across five sections, completed by students following the interprofessional model-making module. Full item-by-item response data were available for this instrument in the current dataset, allowing genuine item-level reliability analysis (Cronbach's α and corrected item-total correlations), computed directly from the current sample (one participant with missing responses on this questionnaire was excluded listwise, n = 49).

### Table 3 Internal Consistency Reliability — Module Feedback Questionnaire, by Section (N = 49)

| Section | Items (k) | n | Cronbach's α |
|---|---|---|---|
| Section 1 — Overall Module * | 11 | 49 | **0.685** |
| Section 2 — Phase-1 Learning Experience | 2 | 49 | **0.278** |
| Section 3 — Importance of Problem-Solving Models | 6 | 49 | **0.591** |
| Section 4 — Model-Making Process | 7 | 49 | **0.667** |
| Section 5 — Assessment Pattern (partial — 3 of 5 canonical items) ** | 3 | 49 | **0.600** |
| **Module Feedback Questionnaire — Total** | **29** | **49** | **0.805** |

*Note. α = Cronbach's alpha. Section 2 contains only two items; short subscales of this kind are mathematically prone to lower alpha values even when individual items are sound, and this should be interpreted with caution rather than as evidence of poor item quality. * One data column included under Section 1 does not match the wording of its canonical instrument item; see Part C, note 6. ** Section 5 here corresponds to canonical Section F, which has 5 items in the source questionnaire; only 3 are present in the analysed dataset. Canonical Section E (4 items) is not represented in this dataset at all and is not shown in this table; see Part C, note 6.*

## Table 4 Item-Total Statistics — Module Feedback Questionnaire (N = 49)

Corrected item-total correlations (r_it) and Cronbach's alpha if the item were deleted (α−i) are reported for all 29 items, grouped by section.

| Item Text | M | SD | r_it | α−i |
|---|---|---|---|---|
| ***Section 1 — Overall Module **** | | | | |
| The module was well organized and met my expectations. | 4.43 | 0.68 | 0.38 | 0.66 |
| The module was clearly structured. | 4.31 | 0.62 | 0.07 | 0.70 |
| Overall, the module was interesting and an enjoyable way of learning. | 4.45 | 0.65 | 0.07 | 0.70 |
| Assessment methods were appropriate and fair. | 4.22 | 0.62 | 0.48 | 0.64 |
| The module has enhanced my understanding of the subject. | 4.31 | 0.71 | 0.17 | 0.69 |
| Was the size of student groups appropriate? Yes/No. if no, please comment: | 4.49 | 0.62 | 0.37 | 0.66 |
| The module was relevant to my studies and the teaching methods involved were appropriate for learning. | 4.47 | 0.62 | 0.47 | 0.64 |
| The module was supported by adequate library resources. | 3.78 | 0.90 | 0.36 | 0.66 |
| I have participated actively in seminar/tutorial discussions. | 4.22 | 0.69 | 0.50 | 0.64 |
| I have spent sufficient time reading/researching to fulfil the module's problem-solving requirements. | 4.04 | 0.84 | 0.47 | 0.64 |
| I would recommend conducting such modules on different subjects. | 3.92 | 0.79 | 0.36 | 0.66 |
| ***Section 2 — Phase-1 Learning Experience*** | | | | |
| Integrated lectures have been helpful. | 4.43 | 0.71 | 0.17 | — |
| I have attended all the lectures regularly. | 4.37 | 0.93 | 0.17 | — |
| ***Section 3 — Importance of Problem-Solving Models*** | | | | |
| The models have greatly enhanced my learning about this topic. | 4.47 | 0.58 | 0.14 | 0.62 |
| The models enhanced my creative skills. | 4.33 | 0.63 | 0.27 | 0.57 |
| The models developed my problem-solving skills. | 4.35 | 0.60 | 0.56 | 0.44 |
| The models developed my ability to work as a team member. | 4.47 | 0.62 | 0.23 | 0.59 |
| Having participated in the problem-solving model making, I feel confident about tackling unfamiliar problems. | 4.45 | 0.54 | 0.33 | 0.55 |
| I am satisfied with all the metacognition measuring scales used in the module. | 4.20 | 0.71 | 0.46 | 0.48 |
| ***Section 4 — Model-Making Process*** | | | | |
| The understanding and correlation of clinico-radiological, and arthro-kinematic findings before model making was difficult. | 3.57 | 0.71 | 0.27 | 0.66 |
| The application of the well understood findings in creating the problem-solving model was difficult. | 3.37 | 0.86 | 0.37 | 0.63 |
| I had enough time to correlate, understand the patient findings and their application during the model-making process. | 3.63 | 1.03 | 0.25 | 0.67 |
| The model making process was too heavy. | 3.14 | 1.12 | 0.35 | 0.64 |
| The model making process was overly theoretical and abstract. | 2.49 | 0.94 | 0.52 | 0.59 |
| There was a lot of pressure on me to do well. | 2.71 | 1.10 | 0.48 | 0.60 |
| The sheer amount of work to go through meant that you can't understand it all thoroughly. | 2.98 | 0.83 | 0.43 | 0.62 |
| ***Section 5 — Assessment Pattern (partial — 3 of 5 canonical items) ***** | | | | |
| The questionnaire on metacognition skills were meticulously designed, and they could accurately evaluate my thinking processes. | 4.12 | 0.78 | 0.57 | 0.26 |
| The questionnaire on creativity and self-regulation was precisely directed to evaluate my creative skills. | 4.08 | 0.73 | 0.48 | 0.41 |
| To excel in model making, proficiency in artistic and craft skills is essential. | 4.02 | 0.90 | 0.23 | 0.78 |

*Note. r_it = corrected item-total correlation (item score correlated with the sum of the remaining items in its section); α−i = Cronbach's alpha for the section with that item removed (not computable, shown as —, for the 2-item Section 2). M and SD are reported on the original 1–5 response scale.*

## Part C: Acknowledgement of Limitations

1. Content validity (expert-panel I-CVI) was not re-derived for the current manuscript. For SSCS and MCPS, content and construct validity are documented in their original publications (see References). The GMS and Teamwork Satisfaction Scale were adapted from Chao, Chiu, Tsai, et al. (2024); content validity (I-CVI = 1.00 across all items) and full item-level reliability for the adapted GMS and the adapted MAI in this study population were established in the companion Phase 2 validation study (Charmode et al., under review) using an independent three-expert review panel, and are referenced here rather than repeated, since a new expert review was not conducted for this phase. The Model Scoring Scale is a self-designed faculty rubric without a published content-validity index; its scoring criteria are reproduced in full in Supplementary File 6 (Compiled Questionnaires, Instrument 5).

2. Item-level raw responses for SSCS, MCPS, GMS, and the adapted MAI are not retained in the current study's master dataset (only subscale and total summary scores are available), which prevented recalculation of item-total correlations or factor loadings for these instruments in the current sample. Similarly, only the total Model Scoring Scale score per model/team is retained, not the rating on each of its 10 individual criteria, so inter-rater or criterion-level reliability could not be computed for this rubric.

3. The MAI composite-level alpha reported in Table 2 (α = 0.68, based on 8 sub-dimension scores) is an exploratory, lower-resolution estimate and is not equivalent to, and should not be compared directly with, the 52-item alpha (α = 0.95) reported in the Phase 2 validation.

4. One of 50 enrolled students was excluded from all analyses due to absence at data collection, yielding a final analytic sample of N = 49, consistent with the companion Phase 2 study.

5. As in the companion Phase 2 study, the GMS and adapted MAI were treated as independent measures; no confirmatory factor analysis was run on either instrument in the current phase.

6. Data-correspondence flag for the Module Feedback Questionnaire (Tables 3–4): the version of this instrument compiled with full item wording and citation (Supplementary File 6 — Compiled Questionnaires) contains six sections (A–F, 31 Likert items total). The raw master dataset used for the reliability analysis in this file contains only five of those sections and 29 items. Specifically: (a) the item in the data column used as "Section 1, item 6" does not match the wording of the canonical Section A item 6 ("The module has developed metacognitive, collaborative, and problem-solving skills.") — the data column instead contains responses to the open-ended question "Was the size of student groups appropriate?", which belongs elsewhere in the instrument; (b) canonical Section E ("Collaborative session with the interprofessional team," 4 items) is entirely absent from the master dataset; (c) canonical Section F ("Assessment pattern," 5 items) is represented by only 3 of its 5 items in the master dataset (the items on whether the team tested memorisation versus understanding, and whether assessment focused on the model's functional working, are missing). The Cronbach's alpha and item-total statistics in Tables 3 and 4 are computed correctly from the columns that were present, but the item identified as "Section 1, item 6" should be verified and relabelled or excluded, and Sections E and F should be reconciled against the source data collection instrument before these results are reported in a manuscript.

7. Additional administered instruments not reported in detail. As noted in the main manuscript, additional instruments administered as part of the registered project (the Readiness for Interprofessional Learning Scale [RIPLS], the Creative Self-Regulation [CSR] scale, the Teamwork Satisfaction scale, and individual model-rubric scores) did not show statistically significant changes and are therefore not reported in detail.

8. **Registered outcomes beyond the scope of this report.** The intermediate- and long-term outcomes registered with CTRI (retention of creative problem-solving skills in the second professional year and downstream patient-care outcomes) were beyond the scope and timeframe of this report and are not assessed here.

9. **Blinding as recorded in the trial registry.** Although the trial registry records participant and investigator blinding, in practice blinding applied only to the independent scoring of the model-making rubric; given the nature of this single-arm educational intervention, participants and facilitators were not blinded.

## References


1. Chao, C. T., Chiu, Y. L., Tsai, C. L., et al. (2024). Identifying group metacognition associated with medical students' teamwork satisfaction in an online small group tutorial context. BMC Medical Education, 24, 1114. https://doi.org/10.1186/s12909-024-06116-4
2. Charmode, S. H., Mohammed, C. A., Salam, A. A. A., Mehra, S., & Gupta, S. (under review). Team collaboration and group metacognition in enhancing problem-solving among Indian medical undergraduates: A quasi-experimental mixed-methods study. Manuscript submitted for publication.
3. Karwowski, M., & Lebuda, I. (2018). Measuring creative self-efficacy and creative personal identity. The International Journal of Creativity & Problem Solving, 28(1), 45–57.
4. Lynn, M. R. (1986). Determination and quantification of content validity. Nursing Research, 35(6), 382–385.
5. Nunnally, J. C. (1978). Psychometric theory (2nd ed.). McGraw-Hill.
6. Polit, D. F., & Beck, C. T. (2006). The content validity index: Are you sure you know what's being reported? Research in Nursing & Health, 29(5), 489–497.
7. Schraw, G., & Dennison, R. S. (1994). Assessing metacognitive awareness. Contemporary Educational Psychology, 19(4), 460–475.
8. Urban, K., & Urban, M. (2023). How can we measure metacognition in creative problem-solving? Standardization of the MCPS scale. Thinking Skills and Creativity, 49, 101345.

# SUPPLEMENTARY FILE S8

## *Qualitative Coding Framework, Credibility Check, and Audit Trail*

This supplementary file documents the rigor of the qualitative thematic analysis conducted as part of the CPSSM (Creative Problem-Solving Skills through Metacognition) study, including the inter-rater reliability process, the final coding framework, credibility and trustworthiness checks, the audit trail, reflexivity statement, and ethical conduct of data collection, consistent with the methods described in the main manuscript's Data Analysis subsection.

## Part A: Inter-Rater Reliability

Qualitative thematic coding: three independent raters (the principal investigator and two external clinician educators) independently coded a random 20% subset of the open-ended student responses.

- Initial agreement: 82%.
- Final consensus: all discrepancies were resolved through discussion, yielding Cohen's κ = 0.87, indicating strong inter-rater reliability.

Quantitative rubric scoring (reported here for completeness, distinct from the qualitative coding above): model-making rubric scores were assigned through blinded scoring by one independent faculty rater, separate from the qualitative coding team, yielding an intraclass correlation coefficient (ICC) = 0.88 against a second scorer.

## Part B: Coding Framework

Thematic analysis followed Braun and Clarke's six-phase approach: familiarisation with the data, generation of initial codes, searching for themes, reviewing themes, defining and naming themes, and producing the final report. A total of 452 initial codes were generated from the open-ended responses and clustered into 5 themes and 12 subthemes. Representative quotations illustrate each subtheme below.

### Table S8.1 Final Coding Framework

| Subtheme | Example Codes | Representative Quotes | Credibility Checks |
|---|---|---|---|
| **Theme: Collaborative Dynamics** | | | |
| Team support | teammate support, coordination, leadership | "Teamwork in our group, roommate support." / "Teamwork & time management; leader helped cooperation." | Negative cases documented: some members reported as absent or uncooperative. |
| Motivation | encouragement, drive to succeed | "Motivation to win." / "Motivation by team & friends." | Member-checking confirmed motivational factors as valid. |
| **Theme: Resource & Process Challenges** | | | |
| Resource constraints | materials, availability, logistics | "Resources were difficult." / "Collection of raw material for the model." | Negative case documented: "lack of resources in market, difficulty of folding LED strips." |
| Time pressure | deadlines, workload | "Deadline." / "More time required, it disturbed routine schedule." | Member-checking: 5 respondents validated time pressure as a major challenge. |
| **Theme: Learning Gains** | | | |
| Creativity & problem-solving | innovation, model design | "Creativity increased, teamwork, presenting model." / "Problem-solving skill." | Confirmed by 10% member-checking sample. |
| Confidence & communication | presentation, social skills | "Presenting about my team model, I felt my confidence increased." / "Improved communication skill." | Negative cases documented: some students reported anxiety or overdemanding teammates. |
| **Theme: Metacognitive Development** | | | |
| Strategy planning | planning, leadership, reflection | "Proper planning." / "Leadership challenge." | Audit trail shows coding consensus. |

| Subtheme | Example Codes | Representative Quotes | Credibility Checks |
|---|---|---|---|
| Self-monitoring shifts | awareness, regulation | “Metacognition.” / “Depending on others & their ideas in group activity can lessen your burden.” | Member-checking confirmed metacognitive awareness as a recurring theme. |
| **Theme: Module Design Feedback** | | | |
| Strengths | organization, innovation | “The module is very organized, well prepared & managed.” / “Interesting, innovative and new to me.” | Audit trail: Excel coding logs retained. |
| Areas for improvement | lectures, time, resources | “Integrated lecture sometimes not understood at all.” / “Model making is very time-consuming.” | Negative case documented: “too theoretical, very superficial in terms of learning.” |

*Note. Quotations are reproduced verbatim (with minor spelling normalisation) from students’ own open-ended responses collected as primary study data; as participant-generated data rather than third-party published material, no copyright restriction applies to their reproduction here.*

## Part C: Credibility and Trustworthiness Checks

- Inter-rater reliability (qualitative coding): Cohen’s κ = 0.87 (principal investigator and two external clinician educators).
- Inter-rater reliability (model-grading rubric): ICC = 0.88 (independent faculty rater).
- Negative/discrepant cases: documented and retained rather than discarded (e.g., resource shortages, uncooperative team members, reports of anxiety or overdemanding teammates).
- Member-checking: approximately 10% of quotations (≈ 5 students) were shared back with respondents for validation; no major discrepancies were raised.
- Audit trail: maintained via the coded master Excel dataset, decision logs from consensus meetings, and successive coding iterations (see Part D).

## Part D: Audit Trail Documentation

- Source data file: Master_chart_wrt_Dr__Sundip_Charmode_Qualitative_data.xlsx (qualitative open-ended responses, as referenced in the main manuscript's Supplementary File S5).
- Coding logs: Excel worksheet recording all 452 initial codes and their clustering into 5 themes and 12 subthemes.
- Decision logs: records (Word documents) of consensus meetings among the three coders, documenting how discrepancies were discussed and resolved.
- Validation records: member-checking notes documenting respondent confirmation of coded themes.

## Part E: Reflexivity

The Principal Investigator (PI), as a faculty anatomist who led the design and delivery of the CPSSM module, acknowledges an insider positionality as a potential source of interpretive bias in the qualitative analysis.

- Bias was minimised through blinded rubric scoring (ICC = 0.88), standardised coding protocols, and triangulation with two independent external clinician-educator raters.
- A reflexivity journal was maintained throughout analysis to document interpretive decisions and cross-check these against the coding consensus reached with the other raters.
- Interpretive balance was supported by comparing qualitative findings against the quantitative outcomes, examining points of convergence and divergence (see manuscript Results and Discussion).
- Discrepant interpretations among coders were resolved through discussion to consensus, rather than being deferred to the PI’s individual reading of the data.

## Part F: Ethics and Voluntary Participation

Participation in all study assessments (questionnaires, the model-making task, and the feedback questionnaire) was voluntary, conducted under the ethics approval and trial registration described in the main manuscript, and supported by signed informed consent from each participating student.

- Uptake rate: 98% (49 of 50 enrolled students completed the intervention and assessments; one student was absent throughout).
- No coercion was reported; students retained the right to withdraw at any stage without academic consequence.
- Ethical approval: Institutional Ethics Committee (IEC), AIIMS Rajkot (Protocol ID: D/NF/74/2024; O.W. No.: AIIMS Rajkot/6th IEC/47/2025, dated 4 February 2025).
- Trial registration: Clinical Trials Registry – India (CTRI/2025/02/080559), prospectively registered 14 February 2025.

## Part G: Conclusion

This supplementary file demonstrates the rigor underlying the qualitative analysis conducted within the CPSSM study. The coding framework, inter-rater and member-checking credibility checks, documented audit trail, and reflexivity statement together provide transparency into how the qualitative findings reported in the main manuscript were generated, supporting their trustworthiness and reproducibility.

# Supplementary file 9

# MCQ design and Classification in Pre- and Post-test

# (10 clinical case scenarios used in CPSSM module)

## 1. Introduction

Pre and post-tests were developed for ten clinical anatomy case scenarios used within the CPSSM module to evaluate baseline knowledge and subsequent learning outcomes of 49 study participants. The modules addressed were Liver, Inguinal canal, pelvic diaphragm, femoral triangle, Arches of foot, Knee joint, Larynx, Tympanic cavity, Ventricular system and CSF circulation, and Facial nerve.

This document reports the MCQ design strategy, the types of questions employed, and the distribution between recall-based and application-based items, of 20 questions used per module for both pre and post-tests.

## 2. Methods of Classification

MCQs were classified using established educational frameworks:

- Bloom's Taxonomy:
    i. Knowledge /comprehension → Recall-based items.
    ii. Application/Analysis → Application-based items.
- Miller's Pyramid of Clinical Importance:
    i. Knows → Recall-based items.
    ii. Knows how → Application-based items.

This ensured that the assessments measured both **foundational knowledge** and **clinical reasoning skills**.

3. MCQ Design Characteristics

- Format : All items were constructed as single -best answer MCQs with four options.
- Stem types:
    i. Direct factual recall.
    ii. Negative stem (e.g. Which statement was INCORRECT?)
    iii. Clinical Vignette (patient scenario)
    iv. Structure – function correlation
    v. Image /diagram-based questions (e.g. liver segments, foot arches, knee ligaments)
    vi. Matching -type questions (e.g. facial nerve branches, CSF pathways)

- Integration: The questions were linked to surgical procedures, pathological conditions, and clinical correlates.
- Cognitive level: Items ranged from recall of factual knowledge to application of anatomical principles in clinical contexts.

4. **Distribution of MCQ Types (Pre-test, 20 Questions per Module)**

| Module | Recall-based | Application-based | Recall % | Application % |
|---|---|---|---|---|
| **Liver** | 12 | 8 | 60% | 40% |
| **Inguinal canal** | 11 | 9 | 55% | 45% |
| **Pelvic diaphragm** | 13 | 7 | 65% | 35% |
| **Femoral triangle** | 10 | 10 | 50% | 50% |
| **Arches of foot** | 12 | 8 | 60% | 40% |
| **Knee joint** | 11 | 9 | 55% | 45% |
| **Larynx** | 13 | 7 | 65% | 35% |
| **Tympanic cavity** | 14 | 6 | 70% | 30% |
| **Ventricular system and CSF circulation** | 15 | 5 | 75% | 25% |
| **Facial nerve** | 12 | 8 | 60% | 40% |
| **Total (Pre-test)** | **123** | **77** | **61.5%** | **38.5%** |

5. **Distribution of MCQ Types (Post-test, 20 Questions per Module)**

| Module | Recall-based | Application-based | Recall % | Application % |
|---|---|---|---|---|
| **Liver** | 13 | 7 | 65% | 35% |
| **Inguinal canal** | 12 | 8 | 60% | 40% |
| **Pelvic diaphragm** | 14 | 6 | 70% | 30% |
| **Femoral triangle** | 11 | 9 | 55% | 45% |
| **Arches of foot** | 13 | 7 | 65% | 35% |
| **Knee joint** | 12 | 8 | 60% | 40% |
| **Larynx** | 14 | 6 | 70% | 30% |
| **Tympanic cavity** | 15 | 5 | 75% | 25% |
| **Ventricular system and CSF circulation** | 16 | 4 | 80% | 20% |
| **Facial nerve** | 13 | 7 | 65% | 35% |
| **Total (Post-test)** | **133** | **67** | **66.5%** | **33.5%** |

6. Comparative Analysis

- Pre-tests contained a broader mix of formats (including matching-type and figure-based items), with recall at 61.5% and application at 38.5%.
- Post-tests streamlined the design, focusing on single-best answer and clinical vignette formats, with recall at 66.5 % and application at 33.5%.
- This shift reflected a deliberative emphasis on knowledge consolidation after module completion, while still retaining a meaningful proportion of application-based items to tests clinical reasoning.

**7. Pedagogical Implications**

- The majority of questions in both pre- and post-tests were recall-based, ensuring participants demonstrated foundational anatomical knowledge.
- Application-based items were consistently included to embed anatomy in clinical scenarios and test reasoning.
- The inclusion of diverse formats in pre-tests promoted diagnostic assessment, while the streamlined post-tests emphasized outcome evaluation.
- This design strategy aligned with Bloom's taxonomy and Miller's pyramid, ensuring assessment of both knows and knows how levels of competence.

**8. Conclusion**

The MCQ design across these ten clinical case scenario modules used in CPSSM Module demonstrated a **structured, multi-type approach** to assessment. Pre-tests incorporated recall, application, matching, and figure-based items to establish baseline knowledge. Post-tests streamlined the format while maintaining a balance between recall and application, effectively measuring both **knowledge retention** and **clinical reasoning skills**. This methodological documentation supports the validity of the assessment strategy and provides transparency for replication in future medical education research.
